# Network dynamics of eukaryotic LTR retroelements beyond phylogenetic trees

**DOI:** 10.1186/1745-6150-4-41

**Published:** 2009-11-02

**Authors:** Carlos Llorens, Alfonso Muñoz-Pomer, Lucia Bernad, Hector Botella, Andrés Moya

**Affiliations:** 1 Institut Cavanilles de Biodiversitat i Biologia Evolutiva (ICBIBE), Universitat de València, Paterna, Valencia, Spain; 2 Biotechvana, Parc Científic, Universitat de València, Paterna, Valencia, Spain; 3 Departamento de Sistemas Informáticos y Computación (DSIC), Universitat Politècnica de València, Valencia, Spain; 4 Área de Paleontología, Dpto. Geología, Universitat de València, Paterna, Valencia, Spain; 5 Centro Superior de Investigación en Salud Pública (CSISP), Valencia, Spain; 6 CIBER de Epidemiología y Salud Pública (CIBERESP), Barcelona, Spain

## Abstract

**Background:**

Sequencing projects have allowed diverse retroviruses and LTR retrotransposons from different eukaryotic organisms to be characterized. It is known that retroviruses and other retro-transcribing viruses evolve from LTR retrotransposons and that this whole system clusters into five families: *Ty3/Gypsy, Retroviridae, Ty1/Copia, Bel/Pao *and *Caulimoviridae*. Phylogenetic analyses usually show that these split into multiple distinct lineages but what is yet to be understood is how deep evolution occurred in this system.

**Results:**

We combined phylogenetic and graph analyses to investigate the history of LTR retroelements both as a tree and as a network. We used 268 non-redundant LTR retroelements, many of them introduced for the first time in this work, to elucidate all possible LTR retroelement phylogenetic patterns. These were superimposed over the tree of eukaryotes to investigate the dynamics of the system, at distinct evolutionary times. Next, we investigated phenotypic features such as duplication and variability of amino acid motifs, and several differences in genomic ORF organization. Using this information we characterized eight reticulate evolution markers to construct phenotypic network models.

**Conclusion:**

The evolutionary history of LTR retroelements can be traced as a time-evolving network that depends on phylogenetic patterns, epigenetic host-factors and phenotypic plasticity. The *Ty1/Copia *and the *Ty3/Gypsy *families represent the oldest patterns in this network that we found mimics eukaryotic macroevolution. The emergence of the *Bel/Pao, Retroviridae *and *Caulimoviridae *families in this network can be related with distinct inflations of the *Ty3/Gypsy *family, at distinct evolutionary times. This suggests that *Ty3/Gypsy *ancestors diversified much more than their *Ty1/Copia *counterparts, at distinct geological eras. Consistent with the principle of preferential attachment, the connectivities among phenotypic markers, taken as network-represented combinations, are power-law distributed. This evidences an inflationary mode of evolution where the system diversity; 1) expands continuously alternating vertical and gradual processes of phylogenetic divergence with episodes of modular, saltatory and reticulate evolution; 2) is governed by the intrinsic capability of distinct LTR retroelement host-communities to self-organize their phenotypes according to emergent laws characteristic of complex systems.

**Reviewers:**

This article was reviewed by Eugene V. Koonin, Eric Bapteste, and Enmanuelle Lerat (nominated by King Jordan)

## Background

Mobile genetic elements (MGEs) are abundant selfish components of living organisms that have helped sculpt the complexity and size of their host genomes over the course of evolution [1]. In particular, retroelements (retrotransposons and retroviruses) constitute a widespread super-family of MGEs that are reverse-transcribed into a double-stranded DNA copy for insertion into their host genomes. Retroelements can be divided into four major systems: the long terminal repeat (LTR) retroelements; the tyrosine recombinase (YR) retroelements; the non-LTR retroelements; and the Penelope retrotransposons (for a review, see [2]). Our study is about LTR retroelements. These encompass the broad panoply of LTR retrotransposons and retroviruses circulating among plants, fungi and animals and can be classified into four major groups or families: the *Ty3/Gypsy*, the *Retroviridae*, the *Ty1/Copia *and the *Bel/Pao *families (see [2]). LTR retroelements share ancestry not only with other retroelement systems, but also with diverse host genes and with a variety of viruses [2]. The most obvious examples of viruses evolutionarily related to LTR retroelements are plant caulimoviruses (*Caulimoviridae*). These are circular double-stranded DNA pararetroviruses that replicate in plants via a RNA intermediate evolved from LTR retroelements [3]. Although caulimoviruses do not have LTRs, they have been included in this work because their study is essential for understanding the deep history of LTR retroelements. For simplicity's sake, we will use the term "LTR retroelement system" throughout the rest of this paper to refer collectively to all families investigated, including caulimoviruses.

An important aim of the post-genomic era is to classify all MGEs and viruses inhabiting (or circulating in) living organisms to understand their role not only as pathogenic agents but also as vectors of evolution. Regarding LTR retroelements, phylogenetic analyses usually show how families split into multiple distinct lineages (clades and genera), but it is yet unclear how deep evolution occurred in this system. The traditional notion in the origin and evolution of the distinct families is monophyletic, but such an assumption has been challenged by increasing evidence suggesting that natural evolution can proceed by gradual and vertical means, but also through distinct modular, saltatory, and reticulate events [3-14]. In general, phylogenetic analyses accumulate systematic and sampling errors when attempts are made to force natural evolution into a tree-like mode. In the words of WF Doolittle [15] - molecular phylogeneticists will have failed to find the true tree, not because their methods are inadequate or because they have chosen the wrong genes, but because the history of life cannot properly be represented as a tree. Our research is consistent with this perception. In a previous study [16] we discovered a network of relationships between *Ty3/Gypsy *and *Retroviridae *LTR retroelements on the basis of gag and pol polyproteins. Our results suggested a scenario of polyphyly, whereby we proposed three *Ty3/Gypsy *ancestors in the deep evolutionary history of the *Retroviridae *(that is the three kings hypothesis). Later [17], we found that on the basis of clan AA of aspartic proteases [18], this network extends not only to the *Ty1/Copia, Bel/Pao *and *Caulimoviridae *families but also to other host peptidases, described in both prokaryotes and eukaryotes (on this topic, see also [19,20]). This triggered our interest in investigating the natural history of LTR retroelements, not only as a tree but also as a network system. In this regard, network biology is an emerging field of graph theory that does not provide all the answers to all evolutionary questions, but that offers an appropriate framework for the study of complex systems (for more information on this topic, see [21-24]). Taking this into primary consideration, we performed (and present in this paper) a comprehensive study investigating not only the phylogenetic diversity of LTR retroelements, but also their evolutionary dynamics beyond phylogenetic trees. In particular, we will show how eukaryotic LTR retroelements are not only a phylogenetic system but also a self-organized system of scale-free characteristics, as observed in all complex networks of major scientific and social interest.

## Results and discussion

### Phylogenetic patterns of LTR retroelements based on pol

LTR retroelements known to date can be classified into four major families, namely *Ty3/Gypsy*, *Ty1/Copia*, *Bel/Pao *and *Retroviridae *[2]. These share ancestry with the *Caulimoviridae *[3] through two common polyproteins, gag and pol. Gag usually contains three protein domains called matrix (MA), capsid (CA) and nucleocapsid (NC). Analyses based on this polyprotein are rarely reported because it evolves rapidly. In contrast, pol consists of four protein domains termed protease (PR), reverse transcriptase (RT), ribonuclease H (RH), and integrase (INT). RT shows a robust phylogenetic signal and it is commonly used for inferring the evolutionary history of LTR retroelements (see [2]). The RT evolutionary history comprises multiple distinct phylogenetic patterns and is further supported by other phylogenies inferred on the basis of RH [8], INT [4,25] and PR [17]. However, increasing evidence suggests that this history is accurate only with respect to the clustering of OTUs into lineages (clades and genera) because evolution of transposable elements is in most cases modular (see [5]). Indeed, in attempts to investigate the deep history of LTR retroelements, the internal tree topology usually accumulates systematic errors and shows various important discrepancies depending on the pol protein domain evaluated. Upon this, in a previous approach [26] we found that when LTR retroelement phylogenies are inferred from the concatenation of two or more protein domains, the statistical power of the analysis is increased and diverse (but not all) single-gene discrepancies are corrected. This strategy enables taxonomy levels to be assigned to the different OTUs evaluated and provides an accurate perspective on the multiple distinct phylogenetic patterns of each family. With this aim, we used an alignment concatenation of the PR, RT, RH and INT pol protein domains to infer the phylogeny of each family using the Neighbor Joining (NJ) method of phylogenetic reconstruction [27]. A description of each family follows (for more details about sequences and lineages, see Additional file 1 or the section "Sequences" in Methods).

#### Ty3/Gypsy

**Figure 1:**
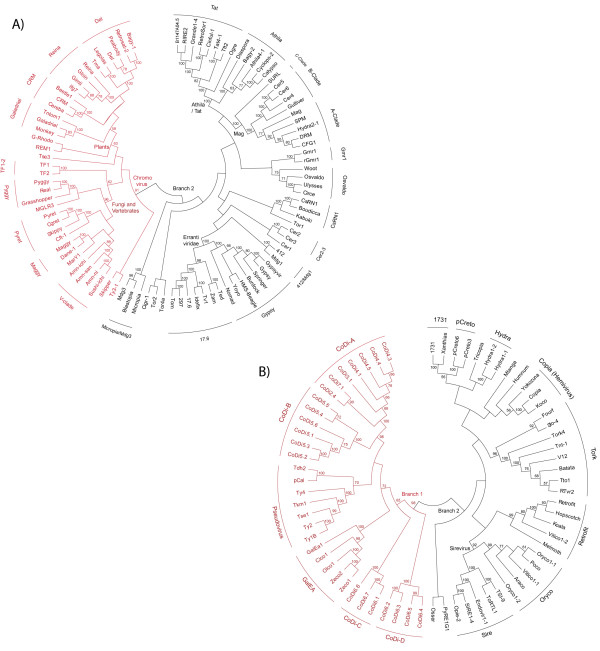
***Ty3/Gypsy *and *Ty1/Copia *phylogenies**. A) Inferred NJ tree of *Ty3/Gypsy *LTR retroelements based on pol. Bootstrap values over 55 are indicated. The tree can be divided into two major branches: one encompassing all chromoviral LTR retrotransposons (in red), the other (Branch 2, in black) encompassing the remaining lineages of LTR retrotransposons and retroviruses. B) Inferred NJ tree of *Ty1/Copia *LTR retroelements based on pol. This tree reveals two major branches, Branch 1 and Branch 2 (in red and black, respectively). For more information about the different sequences used, see the section "Sequences" under Methods.

These elements constitute a family of retroviruses and LTR retrotransposons widely distributed among the genomes of plants, fungi and animals. According to the International Committee on the Taxonomy of Viruses (ICTV), *Ty3/Gypsy *elements were originally classified into two major genera called *Metaviridae *and *Errantiviridae *(see [2]). For some time, this classification has been an important reference in LTR retroelement taxonomy, but it is now considered inconclusive by many authors because sequencing projects have revealed new lineages, updating the original perspective. As shown in Figure 1A, the inferred phylogeny of *Ty3/Gypsy *LTR retroelements reveals two large branches. The first branch (in red) encompasses all chromodomain-INT-containing LTR retrotransposons [4], usually called chromoviruses [28]. This branch includes at least two well-supported clusters of LTR retrotransposons, here named "Plants" and "Fungi/Vertebrates". The cluster of plant chromoviruses splits into six clades detailed in the figure, while that of fungi and vertebrates consists of seven clades. This includes a new clade, which we call V-clade, based on the name suggested by Oliver Piskurek (in personal communication) to describe *Amn*-like [29] and *Sushi*-like chromoviruses of vertebrates, collectively. The chromoviral branch includes other divergent fungal elements, which have unclear classification. The second *Ty3/Gypsy *branch (in black), referred to as Branch 2, encompasses the remaining lineages of non-chromoviral *Ty3/Gypsy *LTR retrotransposons and plant and animal retroviruses.

#### Ty1/Copia

This is a family of retroviruses and LTR retrotransposons abundantly represented in the genomes of plants, fungi and animals. The present ICTV classification [30] of the *Ty1/Copia *into three genera - Pseudovirus, Hemivirus and Sirevirus - has limitations identical to those of *Ty3/Gypsy *LTR retroelements. It was important for understanding the original family but it is not conclusive for managing the currently-available diversity of the *Ty1/Copia *family. As shown in Figure 1B, the inferred phylogeny of *Ty1/Copia *LTR retroelements based on pol reveals two major branches, respectively depicted in red and black. The first branch, which we call Branch 1, is supported by bootstrap and encompasses the original Pseudovirus genus (see [31]) together with a clade called GalEA [32], specifically found in marine bilaterians and all *CoDi*-like LTR retrotransposons described in diatoms. The wide variety of *CoDi*-like elements splits into four clades that we simply name *A*, *B*, *C *and *D*. One of the most exciting aspects of *CoDi*-like elements is that the different elements belonging to clade A encode INTs carrying a putative chromodomain at their C-terminus (see Additional File 2, AF2A). The second *Ty1/Copia *branch encompasses the remaining lineages of LTR retrotransposons and potential retroviruses, including the previously described *Copia*-like hemiviruses and sireviruses.

#### Bel/Pao

**Figure 2:**
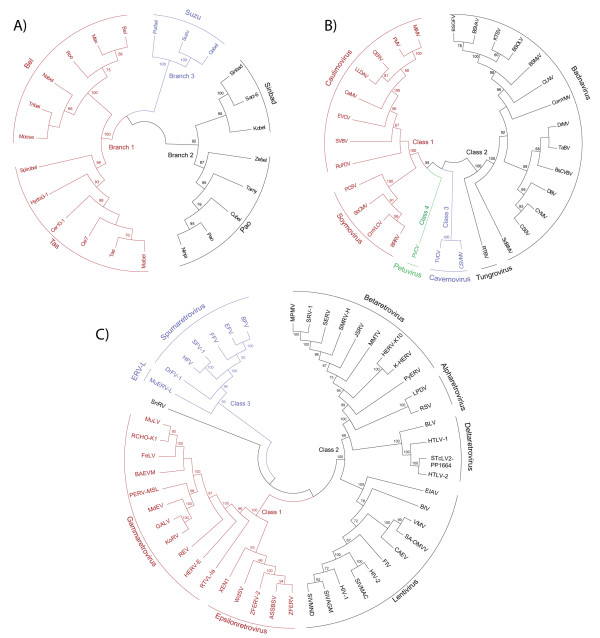
***Bel/Pao, Retroviridae *and *Caulimoviridae *phylogenies**. A) Inferred NJ tree of *Bel/Pao *LTR retroelements based on pol. This tree divides *Bel/Pao *LTR retroelements into five clades collected in three branches, named 1, 2 and 3 (red, black and blue, respectively). B) Inferred NJ tree of caulimoviruses based on pol. The tree is consistent with the six *Caulimoviridae *genera proposed by ICTV [34] but divides them into four classes depicted in red, black, blue and green. C) Inferred NJ tree of *Retroviridae *retroviruses based on pol. This tree shows three classes, namely, 1, 2 and 3 (red, black and blue, respectively), which can be divided into seven genera consistent with ICTV [34]. The tree also includes a taxon representative of ERV-L Class 3 elements as well as a fish retrovirus - SnRV - with unclear location but occupying a place within Class 1 in the LTR retroelement common phylogeny (Additional File 1). For more information, see the section "Sequences" under Methods.

These constitute a family of LTR retrotransposons and retroviruses that have been described to date only in metazoan genomes. According to the ICTV classification, members of the *Bel/Pao *family are called semotiviruses (see [2]). A later study [33] showed that the *Bel/Pao *family can be divided into five clades called *Pao*, *Sinbad*, *Bel*, *Tas *and *Suzu*. Taking a step forward, we show in Figure 2A that on the basis of the inferred pol phylogeny, these five can be collected into three branches that we have numbered 1, 2 and 3. Branch 1 (in red) encompasses two clades - *Tas *and *Bel *- of LTR retrotransposons and retroviruses found in the genomes of cnidarians and protostomes; Branch 2 (in black) includes the *Pao *and *Sinbad *clades of LTR retrotransposons described in protostomes and deuterostomes; Branch 3 (in blue) consists of a single clade called *Suzu*, which includes only LTR retrotransposons that have so far been described exclusively in vertebrates.

#### Caulimoviridae

These comprise a retro-transcribing family of viruses that, in a yet unclear saltatory event, evolved from LTR retroelements to DNA pararetroviruses (see also [3,13]). As shown in Figure 2B, the inferred pol phylogeny of caulimoviruses reveals four branches, which we term "classes" following a viral systematic similar to that of *Retroviridae *retroviruses (discussed below). The four caulimovirus classes may be divided into six lineages - *Caulimo*-, *Soymo*-, *Cavemo*-, *Tungro*-, *Badna*- and *Petuvirus *- according to the genera proposed by ICTV [34] and further corroborated by [3]. Class 1 (in red) includes two genera - *Caulimovirus *and *Soymovirus*; Class 2 (in black) consists of two other genera - *Tungrovirus *and *Badnavirus *- and it is the most abundant branch of *Caulimoviridae *elements; Class 3 (in blue) represents the genus *Cavemovirus*. The phylogeny also includes the *Petunia Vein Clearing Virus *(PVCV), which constitutes the genus *Petuvirus*. This element falls close to Class 1 but occupies the deepest position in the phylogeny (Class 4 in green).

#### Retroviridae

These are a family of retroviruses restricted to vertebrate animals. These retroviruses originally received attention when infectious representatives were characterized in humans. However, it is now known that any LTR retrotransposon capable of recruiting a third ORF envelope gene (*env*) is potentially capable of becoming a retrovirus (*env *is the most basic difference between LTR retrotransposons and retroviruses). Consistent with ICTV [34], the inferred phylogeny of *Retroviridae *based on pol shows seven genera - *Alpha*-, *Beta*-, *Gamma*-, *Delta*-, *Epsilon*-, *Spumaretroviridae *and *Lentiviridae *- that together with *ERV-L *elements we divide into three classes, 1, 2 and 3 (according to [16,35] and references therein). As shown in Figure 2C, Class 1 comprises gamma- and epsilonretroviruses; Class 2 includes lentiviruses, delta-, alpha- and betaretroviruses; and Class 3 encompasses spumaretroviruses and *ERV-L *elements. This analysis includes an element called *Snakehead retrovirus *(SnRV) [36], which has unclear classification; but the common LTR retroelement phylogeny (shown in Additional file 1) places this sequence within Class 1. Finally, the analysis also shows a *Spumaretroviridae*-like element that we found in genome of *Danio rerio *and that we call *Danio rerio Foamy Virus Type 1 *(DrFV-1). This is a complex retrovirus displaying the typical gag and pol ORFs of LTR retroelements and three additional ORFs that show no similarity to any sequence currently known (including *env*). DrFV-1 is taxonomically important because; 1) it is unclear if it is a true retrovirus carrying a highly divergent *env *plus two accessory or additional genes or a LTR retrotransposon just carrying three additional genes; 2) its presence in *Danio rerio *suggests that spumaretroviruses are more widely distributed than previously thought. In Additional file 2 AF2B, we show diverse sequence comparisons using gag and pol ORFs of DrFV-1 as queries against the search of taxonomic HMMs available at the Gypsy Database (GyDB) [26]. These show that DrFV-1 is a spumaretrovirus, but also an intermediate sequence among the *Retroviridae *and other LTR retroelement families.

### What does the post-genomic era suggest about LTR retroelement macroevolution?

**Figure 3:**
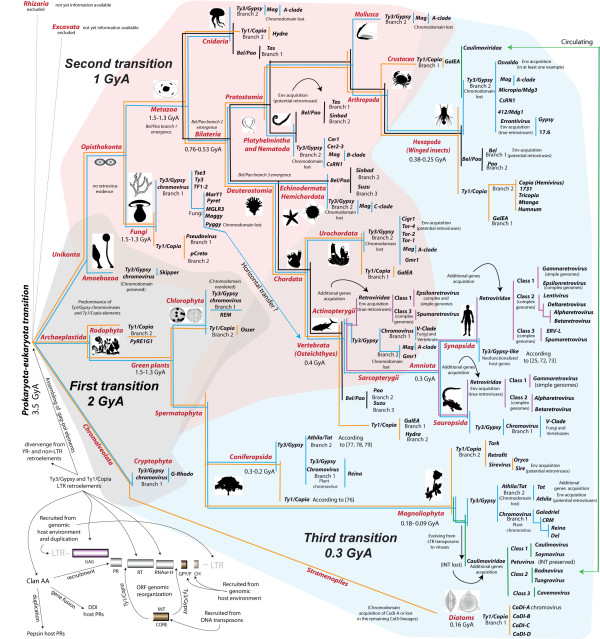
**Evolutionary history of LTR retroelements superimposed over the tree of eukaryotes**. The tree of *Ty3/Gypsy *LTR retroelements is depicted in blue; that of *Ty1/Copia *elements in yellow; *Bel/Pao *in black; *Retroviridae *in violet; and *Caulimoviridae *in green. This set of trees together defines a natural network constrained by the tree of eukaryotes. The figure includes information about diverse reticulate events in the evolutionary history of LTR retroelements. The framework has been divided into three major transitions to elucidate the natural histories of the distinct LTR retroelement communities on the basis of the most likely ages of their hosts, calibrated by previous molecular estimations. The first transition is emphasized in light grey, the second in light red, and the third in light blue.

Phylogenetic analyses help to classify the diversity of LTR retroelements by assigning taxonomy levels to each family. For evaluating the whole system, Additional file 1 shows the inferred phylogeny of the 268 LTR retroelements investigated based on pol using the NJ method of phylogenetic reconstruction. This tree reports a trichotomy that separates the five evaluated families into three major clusters - *Ty1/Copia*, *Bel/Pao *and *Ty3/Gypsy-Caulimoviridae-Retroviridae *(TCR). The latter suggests an ancestral node common to three families that probably corresponds to the *Ty3/Gypsy *family in light of its wide distribution. While this evidences an evolutionary history mainly driven by gradual means, LTR retroelement trees often show branching variations in the internal topology, due to diverse reticulate processes. These may be due to; A) the different rates of evolution among the distinct retroelement protein domains (i.e. modularity [4,5]); B) the incidence of natural mechanisms such as gene recruitment [37], genome rearrangement [6,7] and recombination [8-10]; C) horizontal transfer, caulimoviruses and exogenous retroviruses (and more rarely, endogenous retroviruses and many LTR retrotransposons) usually spread via infection or by horizontal means [11,12,14]; D) saltatory evolution, caulimoviruses are DNA viruses evolved from LTR retroelements (see [3,13]). These processes support an alternative scenario more appropriately described as a network than as a tree (see the phylogenetic networks shown in Additional file 2 AF2C). In this regard, in a previous study [16] we explored the *Ty3/Gypsy *origins of *Retroviridae *retroviruses comparing the *Ty3/Gypsy *family with the three *Retroviridae *classes 1, 2 and 3. We found a network whereby *Ty3/Gypsy *lineages of plants and fungi, such as *Tat *and *Athila *elements and chromoviruses, can be related to the *Retroviridae *Class 1. In turn, other *Ty3/Gypsy *lineages of insects, such as *Micropia/Mdg3 *clade and errantiviruses, can be related to Classes 2 and 3. In light of this polyphyletic scenario we proposed the three kings hypothesis [16], according to which the three *Retroviridae *classes can potentially be tracing three *Ty3/Gypsy *ancestors, emerged at different evolutionary times (see, Additional file 2 AF2C). Therefore, while we can accept that the *Retroviridae *evolved from the *Ty3/Gypsy *family, the way in which their distinct lineages appeared or can be related with the *Ty3/Gypsy *family, is probably not monophyletic. This perspective stimulated our interest in evaluating the evolutionary dynamics of the five LTR retroelement families as a whole system. Upon this, sequencing projects continuously reveal multiple distinct communities of LTR retroelements overlapping in the genome of a single eukaryotic host (see [28,38-40]). It is well known that eukaryotes usually transmit their retroelement communities via germ lines (i.e. by vertical means), except in the case of exogenous retroviruses and caulimoviruses which spread horizontally although only within the boundaries of the same biological supergroup. In other words, the host distribution of the distinct LTR retroelement communities is fairly vertically at the phylum level. Taking this into account, we superimposed the phylogenetic patterns of the LTR retroelement system over the tree of eukaryotes to investigate their macroevolutionary history, at distinct evolutionary times (Figure 3). Although we assume five major supergroups of eukaryotic hosts (Excavata, Rhizaria, Archeoplastida, Chromalveolata and Unikonta, according to [41]) no information about LTR retroelements in Excavata and Rhizaria has been published yet (as far as we know). Therefore, the framework depicted in Figure 3 focuses only on the host distributions of LTR retroelements described in Archeoplastida, Chromalveolata and Unikonta. These were divided in three major transitions by assuming times of emergence, according to the age and macroevolution patterns of their hosts, calibrated by prior molecular estimations and the fossil record. There follows a discussion of the three transitions based on their most parsimonious interpretation.

The first transition traces the most likely root of the LTR retroelement system and covers a long episode in the history of life. This is approximately 1,950-2,170 Millions years ago (Mya) between the Archean and the Proterozoic. The transition runs from the earliest eubacterial fossils and the first unicellular algae to the segregation of eukaryotes into the major supergroups (3,500-1,330 Mya [42-47]). According to this framework, the root of the LTR retroelement system is dichotomous as it consists of two families - *Ty1/Copia *and *Ty3/Gypsy *-, which segregated into two separate phylogenetic patterns. This is probably because of the genomic rearrangement of *int *that differentiates these two. Subsequently, *Ty3/Gypsy *chromoviruses acquired a GPY/F module and a chromodomain. The wide distribution of *Ty3/Gypsy *chromoviruses, not only in algae but also in land plants, amoebae, fungi and animals and chromalveolates, suggests that these constitute the oldest branch of *Ty3/Gypsy *LTR retrotransposons. In Figure 1, we distinguished two branches in regards of the *Ty1/copia *family. Branch 2 spreads among algae, plants, fungi and animals and Branch 1 among fungi, diatoms and marine animals. Also, a prior study [48] describes a *Ty1/Copia *taxon close to the GalEA clade (*Ty1/Copia *Branch 1) in red algae, suggesting that the two *Ty1/Copia *branches probably co-existed with *Ty3/Gypsy *chromoviruses before the split between plants and unikonts (1,550 Mya according to molecular estimations [46,47]). These estimations are older than the earliest predictions of the fossil record (1330-720 Mya [43-45]), but the absence of fossil evidence for the early history of plants, fungi and animals might be because these were probably small or microscopic soft-bodied organisms. Another interesting aspect of this transition is that it seems to link the root of the LTR retroelement system with the evolutionary history of other retroelements. The similarity in both sequence and gag-RT-RH genomic architecture between LTR- and YR retrotransposons suggests an ancient gag-pol form, from which these two systems evolved, probably diverging from other retroelement systems. The segregation of LTR- and YR retroelements seems delineated by several genomic differences. YR retroelements captured a tyrosine recombinase (*yr*) gene (see [49]), while LTR retroelements recruited an *int *gene (probably from DNA transposons [50]) and a clan AA *pr *gene from the host genome [20]. Particularly, the history of clan AA gives additional support to the notion arguing that the history of life diversity is a network of networks, at distinct levels of complexity. This enzyme family spreads in both prokaryotes and eukaryotes and can be divided into two major structural forms; 1) the single-domain ORF typically found in LTR retroelements and several host genes [17,20]; and 2) the two-domain pepsin monomers of eukaryotes classified as the Family A1 at MEROPS [18]. It is commonly assumed that the ancestral clan AA form is the single domain ORF and that pepsins evolved from this form by gene duplication [51]. While this is supported by the wide distribution of the single domain ORF in proteobacteria [17,20] a recent issue has potentially extended the putative origin of pepsins (until now restricted to eukaryotes) by describing homologues in oceanic and plant symbiotic bacteria [19]. This finding re-opens debate about the origin of clan AA but it is, in a way or another, consistent with a major idea; certain genetic units (retroelements and viruses included) of eukaryotes may have evolved from the recombination and assembling of minor genetic elements during the prokaryotic-eukaryotic transition, as proposed in [52,53].

The second transition covers approximately 1,000 million years in the history of life. It should be dated at the segregation of early eukaryotes into their major supergroups (1,330-380 Mya based on [43-45,54]). The transition spans the interval from the Proterozoic to the Devonian Period within the Phanerozoic, during which all major groups of eukaryotes underwent dramatic diversification including key events such as the divergence between primitive animal phyla (e.g. Porifera, Cnidaria, Ctenophora) and higher phyla; the origin of Eumetazoa (1300-940 Mya [54,55]; the Cambrian explosion 540-500 Mya [56]); the rise of vertebrates [57]; the emergence of land plants (probably back to the Ordovician or even the Cambrian Period [58,59]); the origin of plant-fungal interactions [60]; and the divergence between arthropods and priapulids [61]. During this transition, the *Ty1/Copia *and *Ty3/Gypsy *radiated a variety of lineages, and the *Bel/Pao *and the *Retroviridae *families emerged in eukaryotes. To date, there has been no conclusive evidence of *Ty1/Copia *sibling lineages shared by plants and fungi, but *Ty3/Gypsy *phylogenies show two clusters of fungal and plant chromoviruses that are closely related to each other (see Figure 1). Chromoviruses are thus putative markers of ancient interactions between plants and fungi. Interestingly, while chromoviruses spread among vertebrates, they disappear from the genomic record of cnidarians, mollusks, protostomes and basal deuterostomes (according to current data). In contrast, what we find in these organisms are traces of *Ty3/Gypsy *Branch 2. We can therefore assume that *Ty3/Gypsy *Branch 2 elements inhabited bilaterian genomes before the split into protostomes and deuterostomes (761-531 Mya according to [54,62]). Granted this assumption, the oldest *Ty3/Gypsy *lineage in metazoans is probably *Mag *cluster that spreads among cnidarians, protostomes and deuterostomes. Returning to the chromoviruses, it is unclear whether they were driven to extinction in protostomes, echinoderms and urochordates or were horizontally transmitted from plants or fungi to vertebrates. Horizontal transmission seems to be the simplest explanation in view of the strong similarity between *V-clade *and fungal chromoviruses, but if so, the event is at least as ancient as the split of Osteichthyes into actinopterygians and sarcopterygians. This is because *V-clade *is present in fishes such as *Danio rerio *but also in amphibians and amniotes (see also [29,63]). Note that stem osteichthyan occurs in the fossil record in Upper Silurian times, 421 Mya [64], and the oldest unequivocal actinopterygians come from the Lochkovian (410-415 Mya [65]). Moreover, the fossil record indicates that the split between the Actinopterygii and the Sarcopterygii could have taken place between 421 and 416 Mya [62] (consistent with phylogenomic analyses based on expressed sequence tags (ESTs) [66]). The *Ty1/Copia *family is also spread among the genomes of cnidarians, arthropods and deuterostomes, but to our knowledge there is no current evidence of *Ty1/Copia *elements in platyhelminthes and nematodes. Based on the current availability of data, this suggests that *Ty3/Gypsy *ancestors diversified much more than their *Ty1/Copia *counterparts during the history of metazoans. The wide distribution of *Bel/Pao *LTR retroelements among metazoans and their absence from fungi and plants additionally suggests that their origin antedates the split between primitive animals and higher eumetazoans (1300-380 Mya according to [43-45,54]). *Bel/Pao *LTR retroelements are spread among cnidarians, protostomes and deuterostomes and can be related to *Ty3/Gypsy *and *Retroviridae *elements in view of the same *int *order and the presence of a GPY/F module at the C-terminus of INT (see Additional file 2 AF2D). This supports the notion that LTR retrotransposons capable of becoming metazoan retroviruses (*Ty3/Gypsy *Branch 2, and *Bel/Pao *and *Retroviridae *LTR retroelements) might have emerged before the bilateria split into protostomes and deuterostomes (761-531 Mya according to [54,62]). In regards of *Retroviridae *retroviruses, Figure 3 shows that the distribution of *Retroviridae *Class 1 overlaps with that of *V-clade *chromoviruses in *Danio rerio *(and probably in *Salmonidae *species [67]), amphibians and sauropsids ([29]). This suggests that the first true *Retroviridae *taxa probably appeared in the history of vertebrates before the Osteichthyes split into the actinopterygians and the sarcopterygians. The presence of DrFV-1 in *Danio rerio *suggests that *Retroviridae *Class 3 might be as old as Class 1. However, the remaining spumaretroviruses known so far are restricted to synapsids (see [68]). Further analyses are thus required to clarify whether spumaretroviruses also inhabit (or circulate in) the genomes of other fishes, amphibians and sauropsids, to calibrate the origin of *Retroviridae *Class 3.

The third transition covers the period from 330 million years (from the Paleozoic Era) to the present. This is concomitant with diverse events such as the origin of the first gymnosperms (360-248 Mya [59,69]); the split of amniotes into sauropsids and synapsids (330.4-312.3 Mya [62]); the massive radiation of winged insects (380-325 Mya [70]); and the emergence of flowering plants (130-90 Mya [59,71]). These evolutionary events generated new levels of complexity and new ecosystems that probably activated massive radiations of retroviruses and LTR retrotransposons, as well as the emergence of caulimoviruses. Evaluating information of amniotes, we find that the *Ty3/Gypsy *and the *Retroviridae *distributions overlap in the genomes of sauropsids, but there is no evidence of functional *Ty3/Gypsy *elements (nor *Ty1/Copia *and *Bel/Pao*) in mammals where the *Retroviridae *are in turn widely distributed. Despite this, mammalian genomes preserve traces of ancient *Ty3/Gypsy *inhabitants, which are not pseudogenic relics but neo-functionalized host genes evolved from LTR retrotransposons (see, [25,72]). This suggests that *Ty3/Gypsy *LTR retroelements were co-opted by synapsid hosts (mammals and their extinct relatives) and that their extinction as selfish MGEs allowed the three *Retroviridae *classes to spread in these organisms (see also [73]). The two largest *Retroviridae *distributions in amniotes correspond to gamma- and betaretroviruses (see ICTV, online [74]). An important characteristic of *Retroviridae *retroviruses is that almost but not quite all of them carry additional genes. Briefly, gammaretroviruses (Class 1) display the simplest retrovirus organization, typical of *Ty3/Gypsy *and *Bel/Pao *retroviruses; betaretroviruses usually carry one additional gene plus a DUTPase similar to that of various fungal chromoviruses [75]; the remaining genera in classes 2 and 3 show diverse additional genes, which are preserved depending on the lineage (for more information, see [26]). This suggests that gammaretroviruses are probably molecular fossils still preserving the ancient gag-pol-env structure of *Ty3/Gypsy *retroviruses, and that the acquisition of additional genes by the remaining *Retroviridae *genera was an important step in their evolution. As suggested by Katzourakis et al. [68]; "retrovirus accessory genes and mammalian mechanisms of innate immunity will be best understood when considered as the join products of macroevolutionary conflicts played out over a geological scale". When evaluating the known genomes of gymnosperms and angiosperms, we find massive radiations of *Athila/Tat*-like *Ty3/Gypsy *retroviruses and LTR retrotransposons overlapping with their chromoviral counterparts and with several *Ty1/Copia *lineages (see [76-79]). The ancestors of *Athila/Tat *(i.e. all potential the *Ty3/Gypsy *retroviruses and LTR retrotransposons of plants) might have thus emerged before the divergence of conifers and angiosperms (360-248 Mya [59,69]). The most likely age of these lineages is concomitant with the appearance of *Retroviridae *Class 1 gammaretroviruses in amniotes (consistent with the three kings hypothesis). A similar perspective is shown by currently available genomic information on winged insects such as flies, mosquitoes and others. In these organisms we find multiple distinct lineages of *Ty1/Copia, Ty3/Gypsy *and *Bel/Pao *LTR retroelements [39]. In view of the emergence of winged insects (380-325 Mya [70]), these LTR retroelements most probably emerged simultaneously with those of *Retroviridae *classes 2 and 3 in amniotes (again consistent with the three kings hypothesis). Interestingly, caulimoviruses can also be related with *Ty3/Gypsy *LTR retroelements on the basis of the gag-pol region and they show (like the *Retroviridae*) diverse additional genes, which are necessary for their viral life cycle and transmission (see [3,74]). However, this does not relate caulimoviruses with *Retroviridae *retroviruses, as their distinct additional genes cannot be related through function or though similarity. In fact, prior trends relate caulimoviruses to other virus systems based on the common share of the movement protein [13], indicating that the most likely origin of caulimoviruses, was chimeric (an hybrid between *Ty3/Gypsy *retrotransposons and other RNA viruses). It is not yet clear whether the ancestors of caulimoviruses were inhabitants of plants or insects, but their most likely origin was at least simultaneous with the emergence of flowering plants (130-90 Mya [59,71]). This third transition also covers the *Ty1/Copia *LTR retroelements called *CoDi*-like described in diatoms (origins of diatoms dated 164-166 Mya according to [80]). The existence of a *Ty1/Copia *lineage -*CoDi-A *- showing INTs with chromodomain makes of *CoDi*-like elements an interesting case study that merits further attention.

It is too early to draw more specific conclusions, but we think that *CoDi-A *elements might have captured their chromodomain independently from *Ty3/Gypsy *chromoviruses. Note that this feature is a ubiquitous component of many eukaryotic proteins and that no chromodomain has been to date detected in other *Ty1/Copia *elements. This finding is however important in the topic as prior to the present study the status of chromovirus was thought to be restricted to diverse *Ty3/Gypsy *lineages.

### Mapping reticulate (network) evolution patterns

**Figure 4:**
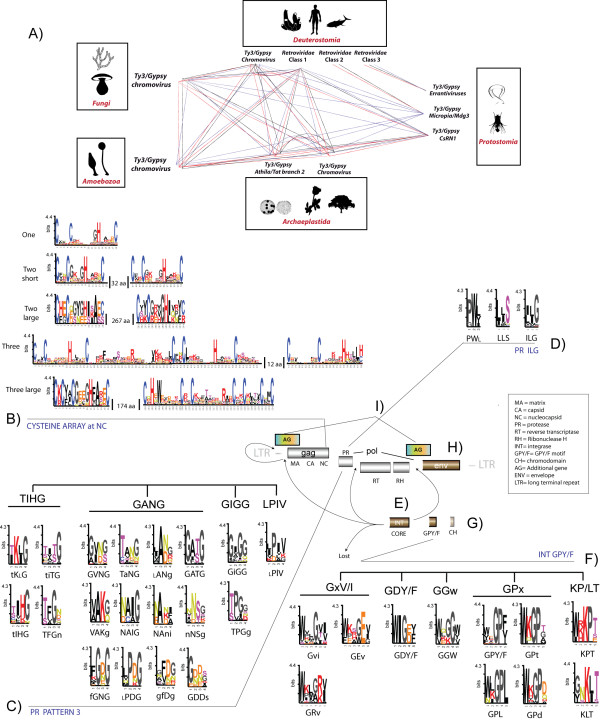
**Intrinsic markers of reticulate evolution**. A) In a previous paper [16] we described a network between diverse *Ty3/Gypsy-Retroviridae *lineages based on similarity, host distributions, and variability of three evolutionary markers. These are the CCHC array in NC (network in red); the sequence motif (namely, pattern 3) defining the structural flap of PR (in black); and the GPY/F motif in the C-terminus of INT (in blue). In view of this network, we performed a deep characterization of these and other markers found not only in the *Ty3/Gypsy *and *Retroviridae *families but also in the *Bel/Pao, Caulimoviridae *and *Ty1/Copia *families. As shown in the figure we considered: B) the number of CCHC arrays in NC; C) all possible variants of pattern 3 in PR; D) the variants of the ILG motif in PR; E) the presence-absence and ORF position of *int*; F) all possible variants of the GPY/F motif in INT; G) the presence-absence of the chromodomain in INT; H) the presence-absence of *env*; I) the presence-absence of additional genes. The figure includes an idealized retroelement genomic reference noting the usual position of each marker.

In the former section we presented an evolutionary framework showing how the history of the LTR retroelement diversity is shaped not only by phylogenetic patterns but also by epigenetic host factors. It is important to stress that no *Retroviridae *and *Bel/Pao *element has been described in the genomes of plants and fungi, and no caulimoviruses have been detected to replicate within animal cells or in their genomes (they act only as vectors). On the more diverse and widely-distributed families such as *Ty3/Gypsy *and *Ty1/Copia *we have an identical perspective. The lineages of these two families described in plants have more-or-less counterparts in fungi and animals but do not inhabit the animal and fungal genomes, and vice versa. The simplest interpretation of this hierarchy is that eukaryotes impose natural barriers to the distribution of LTR retrotransposons, retroviruses and caulimoviruses. This lends support to the idea of an inflationary mode of evolution, whereby we think that the various diversity explosions of eukaryotes induced massive LTR retroelement radiations, as well as the emergence of a network of LTR retroelements. This network co-evolves with the eukaryotic complexity. The first traces of this network are defined by the original genomic reorganization that segregated the *Ty3/Gypsy *and *Ty1/Copia *families into two different phylogenetic patterns. While these two diversified into multiple distinct lineages, our results suggest that the appearance of the *Bel/Pao, Retroviridae *and *Caulimoviridae *families in this network can be related with distinct inflations of the *Ty3/Gypsy *family, at different evolutionary times. This led us to speculate with the idea that *Ty3/Gypsy *LTR retroelements were more successful than their original *Ty1/Copia *counterparts during evolution, because their phenotypic plasticity is higher than that of *Ty1/Copia *LTR retroelements. This might have allowed the *Ty3/Gypsy *family to explore new adaptive strategies during its diversification from which not only lineages, but also families, evolved at distinct geological eras. Following this idea, it is reasonable to assume that the intrinsic evolution of LTR retroelements imprints diversity patterns into a variety of functional retroelement features that derive in the emergence and fixation of molecular phenotypes associated to ancient processes of divergence and convergence. Upon this, in a previous issue we described the existence of a network of relationships between *Ty3/Gypsy *and *Retroviridae *LTR retroelements (discussed in the former section). This network resides not only in sequence similarity and host distributions but also in the duplication-diversification-loss of three molecular features. These are the number of Cys-X2-Cys-X4-His-X4-Cys (CCHC) zinc finger arrays [81] at NC; the sequence flap motif of PR [82]; and the GPY/F motif of the INT module with an identical name [4]. As shown in Figure 4A, the number of CCHC arrays in NC varies depending on the *Ty3/Gypsy *lineage and the *Retroviridae *class (links in red), and both the flap and the GPY/F motifs delineate multiple variant motifs based on sequence polymorphisms preserved similarly (links in black and blue, respectively). These features are lineage-specific markers, suggesting a network scenario of poly- or paraphyly, since they occur not only in *Retroviridae *retroviruses of vertebrates but also in all *Ty3/Gypsy *LTR retrotransposons and retroviruses of plants, fungi and other animals. We have also noted that with respect to the PR marker, we surveyed the diversity of clan AA PRs in a subsequent issue [17]. In that work, we constructed a collection of HMMs as well as a major PR consensus template based on six amino acid patterns and found that not only Ty3/Gypsy and Retroviridae PRs, but also all other PRs, show specific motifs in sequence pattern 3 of this template. This extended the *Ty3/Gypsy-Retroviridae *network to all other LTR retroelements, but only based on PR. From that point onwards, our aim was to investigate all possible markers, not limited to the *Ty3/Gypsy *and *Retroviridae *families but including all other LTR retroelement families. In this particular, we identified eight features that can be classified as gene features (GFs) or as polymorphic amino acid motifs (PAMs). Additional file 3 AF3A provides a detailed "sequence-to-sequence" summary of all these markers and their distinct states, which are also illustrated in Figure 4. A description follows.

Figure 4B shows the CCHC array in NC, which is involved in virion assembly, RNA packaging, reverse transcription and integration processes [83]. This feature is a PAM with six states; zero, one, two or three CCHC arrays (detailed using sequence logos). Arrays based on two and three motifs vary with respect to the size of the sequence between motifs and zero arrays have no logos representation.

Figure 4C describes PR pattern 3 (i.e. the potential or real flap of PR), which confers specificity on the enzyme by carrying important substrate-binding functions [82]. We distinguished 20 states in this PAM including loss of the motif. Nineteen of these were grouped into four major variants: GANG, GIGG, TIHG and LPIV. This follows the nomenclature introduced in [17] and applies an ad hoc criterion of classification. That is, in its major consensus form, the GANG variant, encompasses a variety of motifs related by the predominance of an alanine (or a hydrophobic residue) and an aspartate/asparagine at the second and third positions, respectively. The TIHG variant describes the consensus for diverse motifs that show a threonine at the first position, followed by an isoleucine (or a hydrophobic residue) at the second position and a histidine at the third (or vice versa). The GIGG variant encompasses all sequence motifs with an isoleucine (or another hydrophobic residue) at the second position followed by two glycines. The LPIV variant describes an atypical motif found in only a few Ty1/Copia PRs. Finally, all motifs for which we were unable to resolve a consensus state were combined into a single state considering the loss of the motif. This classification updates the characterization performed in [17] for *Ty1/Copia*, *Bel/Pao *and caulimoviral PRs and revises various *Ty3/Gypsy *motifs resolved in [17].

Figure 4D shows the ILG motif located at the C-terminus of PR. This is one of the most well-preserved features of clan AA PRs [84], and, in the proteinase fold, corresponds with the structural loop that interacts with the catalytic motif (see [17]). On this PAM, we distinguished three states (plus the motif loss) detailed through sequence logos. These are the LLS motif found in *Ty1/Copia *PRs, the ILG motif predominant in all other PRs, and a PWL-like motif preserved by spumaretroviral PRs. Again, motifs for which we resolved no consensus were collected into a single state describing the motif as lost.

Figure 4E details the *int *gene position, on the basis of diverse positional rearrangements in the retroelement genome or its loss. This GF presents five states; first, downstream of *pr*, as in all known *Ty1/Copia *element genomes (see [7]) and *Gmr-1 Ty3/Gypsy *elements [6]; second, downstream to *rh *as usually found in *Bel/Pao *and *Retroviridae *LTR retroelements, and almost but not all *Ty3/Gypsy *LTR retroelements; third, truncated and in reverse frame, as in the REM1 *Ty3/Gypsy *chromovirus, which only preserves the GPY/F module and the chromodomain [85]; fourth, gene loss, as in almost all caulimovirus species except PVCV [86], which shows an INT in direct frame and upstream of gag (this is the fifth state).

Figure 4F describes the GPY/F motif of the module with the same name [4]. The role of the GPY/F module is not yet understood but it is located at the C-terminal end of INT. This trait coordinates the integration of the retroelement into the host genome [87,88]. In regard to the GPY/F motif, we distinguish 13 states. 11 of these have been detailed through sequence logos, and were divided into five variants following a criterion similar to that used for pattern 3 of PR. The GPY/F variants are called GPx, KP/LT, GGw, GDY/F and GxV/I. The GPx variant includes motifs in which a glycine and a proline predominate at the third and fourth positions, respectively. The KP/LT variant collects motifs in which lysine and threonine respectively predominate in the third and fifth positions, while the fourth position is proline or isoleucine/leucine. The GGw variant represents a state based on these three residues. The GDY/F variant encompasses motifs with glycine and aspartate/glutamate (or relatives) at the third and fourth positions, respectively. The GxV/I variant comprises, three motifs commonly based on six residues and usually showing glycine and isoleucine-valine at the fourth and six positions. The two other states considered are motif loss plus the absence of the whole module (as in *Ty1/Copia *INTs and caulimoviruses, which have no INT). This description improves the previous evaluation in [16], which was based only on *Ty3/Gypsy *and *Retroviridae *INTs. In this update we include the GPY/F module we found in *Bel/Pao *INTs (see Additional file 2, AF2D) and revise the previous motif of the Mag-like GPY/F module.

Figure 4G illustrates the chromodomain (i.e. the chromatin organization modifier). The chromodomain is a small protein module involved in chromatin re-modeling and regulation of gene expression (see also [89]), it is found in a variety of host proteins but also at the C-terminus of INT coded by almost all *Ty3/Gypsy *chromoviruses and *CoDi-A Ty1/Copia *LTR retrotransposons (see Additional File 2, AF2A). A previous study showed that *Ty3/Gypsy *chromoviruses probably use this feature for chromatin integration [90]. We consider two states, presence and absence, for this GF.

Figure 4H corresponds to *env*, which potentially confers the capacity to become a retrovirus and is thus necessary for transferring retroviruses cell-to-cell. This is another GF in which we consider the presence or absence of the feature.

Figure 4I considers the additional (accessory) genes of many but not all LTR retroelements. These play a variety of roles in the life cycle and transmission of *Retroviridae *and *Caulimoviridae *viruses. It is now known that several *Ty3/Gypsy *elements belonging to the *Tat *clade and another clade of fungal chromoviruses are also carriers of additional genes (see [31,75]). In this case, we consider the presence or absence of one or more additional genes as a GF.

The above described markers find functional arguments of reticulate evolution in the fact that lineages of distinct families can share not only a marker, but also distinct states of such a marker (for a more exhaustive detail, see Additional file 3). Certainly, the differential preservation of GFs normally derives from ancient processes of gene recruitment, recombination and genomic rearrangement. PAMs have a more intriguing interpretation, as their distinct states could be potentially tracing divergence, convergence, or just random evolution (mutational saturation). In regard to the ILG motif at PR, the LLS and ILG variants make an important taxonomical differentiation between *Ty1/Copia *PRs and the remaining PRs associated to ancient processes of divergence between *Ty1/Copia *and *Ty3/Gypsy *ancestors (with a very few exceptions all *Ty1/Copia *PRs preserve the LLS motif). To evaluate the three other PAMs, we designed a bipartite multigraph model (a graph based on two types of nodes) contemplating three networks analyses that relate the distinct genomic communities of LTR retroelements in eukaryotes with; A) number of CCHC arrays; B) PR pattern 3; C) GPY/F motif. As shown in Additional file 3 AF3B, these networks reveal how the distinct LTR retroelement communities of eukaryotes usually preserve particular PAM states depending on their host distributions. Subsequently, we designed a bipartite multigraph model that contemplates three other networks (Additional file 3, AF3C) relating the three PAMs with the distinct phylogenetic patterns of the LTR retroelement system. This second model illustrates how the multiple distinct states of each PAM are usually redundant among the distinct LTR retroelement families but lineage-specific within them. Certainly, the distribution of PAMs observed in the two discussed graph models cannot be explained as the result of a random association, as the incidence of edges over every PAM node in a random network is approximately the average between distribution edges and nodes [22]. PAMs are thus powerful indicators of divergence, within and among families but we do not dismiss convergent processes in this interpretation because the distinct PAM variants collect motifs and not all sequences belonging to a lineage, in which a variant is predominant, are necessarily carriers of such a variant. This reveals a high degree of phenotypic plasticity whereby evolution is capable of continuously developing, maintaining and recycling functional mechanisms. In other words, whether because of divergence or convergence, it is thus reasonable that PAM variations derive from the fact that the diversity of LTR retroelements is in continuous expansion, not only among families but also within families. We can thus assume that the history of LTR retroelements depends not only on phylogenetic patterns and epigenetic factors, but also on the phenotypic plasticity of LTR retroelements that enables them to adapt these phylogenetic patterns under any epigenetic change of the host environment. This means that the multiple distinct LTR retroelement phenotypes we have investigated are not arbitrary and as a consequence they are limited by the system as a whole. If such an intrinsic feature is in part responsible, to an observable extent, for the complexity of the system, this could result in self-organized, scale-free characteristics.

**Figure 5:**
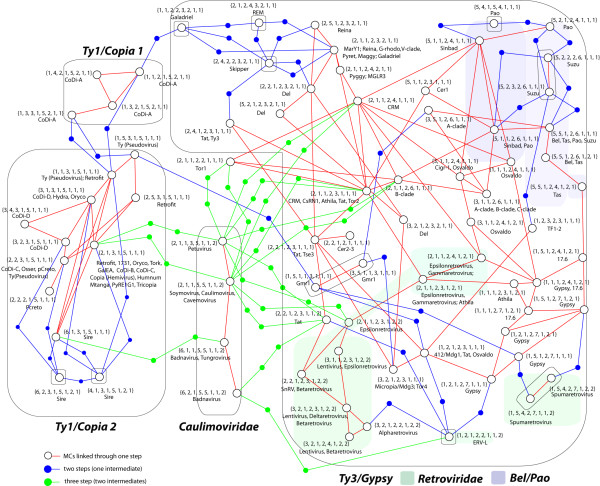
**Phenotypic neighbors' network**. Constructed using the 76 non-redundant MCs (white circles) summarized in Additional file AF3D and 75 intermediates (blue and green dots), as nodes. Each MC is detailed in brackets and accompanied by the names of the lineages that display or share such a phenotypic combination. MCs attach to other MCs on the basis of their phenotypic proximity in single steps. Those sharing 7 of the 8 MC features are connected by a red link. This assumption derives in seven MCs with no links and seven components of phenotypic neighbors, distanced by a single step (in boxes). Components were joined by assuming intermediate phenotypic combinations representing the number of steps needed to relate MCs of distinct components separated by two or three changes. In blue, we join MCs in one component that share 6 of the 8 features with the remaining components. Similarly, in green, we relate MCs sharing 5 of the 8 features. For simplicity's sake, links among MCs of the same initial connected component are not traced as these already have one-step neighbors (for more details, see Additional file AF3E).

Indeed, one of the most important properties of scale-free networks is that the connections between nodes are distributed following a power law. Such a property derives from two facts; 1) real-world networks evolve over time and expand by addition of new nodes; 2) new nodes preferentially attach to nodes that are highly connected (according to [22,24]). This directly results in the small-world phenomenon in which most nodes can be reached from any other in a small number of steps (the threshold is often regarded to be six). Taking this into primary consideration, we aimed to investigate the connectivity between complex phenotypic identities, within and between families. With this aim we combined the eight markers shown in Figure 4 into marker combinations (MCs) to construct a network model based on phenotypic neighbors. In total, we constructed 268 MCs (summarized in Additional file 3 AF3A), one for each LTR retroelement taxon. However, MCs determine phenotypic identities, which can be common to two or more lineages. This means that the number of MCs can be reduced to 76 (detailed in Additional file 3, AF3D), free of redundant combinations. By comparing MCs and detecting common subsets of features, we can trace neighbor relationships, which are shortcuts between lineages of different families. For example, several *Ty3/Gypsy *errantiviruses and *Retroviridae *spumaretroviruses share the following phenotype - "gag with zero CCHC arrays, plus TIHG PR plus INT after RH with GPY/F module carrying a KPT motif"- but differ in the state of the ILG motif and the presence or absence of additional genes. Errantiviruses and spumaretroviruses are related by their MCs by paths of length 2, two steps or trait changes. Following this strategy we constructed the global network of phenotypic neighbors (Figure 5) connecting all MCs, represented by 76 nodes (in white). Single changes are represented by red links, which attach two MCs if they share 7 of the 8 MC features. This produces seven single-node MCs with no links and seven additional components of connected neighbors (detailed in boxes). We then added 2 links and 1 intermediate node among the MCs of components that share 6 of the 8 MC features (in blue). Next, we added 3 links and 2 nodes (in green) to relate those MCs that share 5 of the 8 MC features (for more details, see Additional file AF3E). Connections between MCs in the same component are omitted. In our model, these would be redundant because any MC that can be reached through two or three red links can also be reachable through a blue or a green path, respectively. In doing so, all the initial components are connected in a global network that can be navigated under the relation "one link one change". Intermediate blue and green nodes (inserted between MCs separated by two and three changes, respectively), can be considered as combinations potentially describing lineages not yet characterized or extinguished forms, so that the model allows the evaluation of not only reticulate processes but also putative evolutionary gaps in the history of LTR retroelements. For instance, one can navigate the *Ty1/Copia *family through single changes, except to all *CoDi-A *and two sirevirus MCs. These combinations need two changes to reach other *Ty1/Copia *phenotypes as they differ in the presence of a chromodomain at INT (in the case of the CoDi-A combinations), or in the duplication or triplication of the CCHC array (in the case of the large C-terminus of sirevirus gags). For example, in the CoDi-A case an intermediate node represents any putative sequence halfway between the conventional *Ty1/Copia *and their *CoDi-A *counterparts (for instance a *CoDi-A *element without chromodomain), etc.

The network model of phenotypic neighbors supports the idea of shortcuts (phenotypic proximity) between the *Ty3/Gypsy *and *Ty1/Copia *families. Some *Ty3/Gypsy *combinations are as close to other *Ty3/Gypsy *as to *Ty1/Copia *phenotypes due to; 1) the presence of a chromodomain in *CoDi-A *elements; 2) the *Ty1/Copia*-like INT ORF order of the *Ty3/Gypsy *lineage called *Gmr1*. Another interesting aspect of this network is that it shows multiple distinct identities (MCs sharing the eight markers) and links among different *Retroviridae *and *Ty3/Gypsy *combinations, and among the latter and distinct *Bel/Pao *combinations. The proximity of these three families in terms of phenotypes and ORF organization, suggests that the *Ty3/Gypsy *family diversified much more than the *Ty1/Copia *family, during the course of evolution. Interestingly, the analysis supports the notion of a poly- or paraphyletic relationship between diverse *Ty3/Gypsy *phenotypes and those of *Retroviridae *retroviruses (in agreement with the three kings hypothesis). Similarly, distinct caulimoviral combinations link to each other via a single step but need three steps to connect with other phenotypes (because of differences as diverse as the absence of INT, GPY/F module, env, chromodomain, etc). Almost all the caulimovirus links observed in the network correspond with the changes needed to reach diverse *Ty3/Gypsy *combinations.

Finally, the model of phenotypic neighbors is consistent with the principle of preferential attachment; the high connectivity of a few *Ty3/Gypsy *phenotypes permits the navigation of the whole network step-by-step and from one family into another. Statistical evaluation of the network topology reveals that the connectivities between nodes are power-law distributed (see Additional file AF3E) confirming that the network is free-scale. This type of distribution cannot be explained as the result of a purely random process, as the edge distribution in a random network follows a Poisson distribution with a characteristic value, which is the number of edges divided by the number of nodes (for more details about random networks, see [22]). Moreover, the inference of mean shortest path (the average of the shortest distances between pairs of nodes) gives a value of 5.61 indicating that the network has also small-world characteristics. This is because the model is composed of phenotypic components characterized by the presence of shortcuts between almost any pair of combinations. In fact, the network is constituted by nodes which, in most cases, describe MCs common to two or more lineages. Consequently, if we trace as many links between a pair of nodes as lineages share each represented MC, we can observe that the clustering coefficient follows a power-law (see Additional file AF3E), as typically observed in hierarchical networks (see [22]). The emergence of this hierarchy reinforces the idea that the phenotypic combinations with high connectivity are widely distributed in the system, probably because they represent the most adaptive strategies in the phylogenetic history of LTR retroelements. This fact is not trivial and merits further attention, as it shows that phenotypic plasticity displays both structure and distribution. This structure is determined by properties that do not depend only on phylogenetic phenotype per se or by epigenetic effect of host evolution, but by the capability of the system to self-organize according to emergent laws characteristics of real complex systems.

## Conclusion

The evolutionary history of LTR retroelements can be traced as a time-evolving network that depends on phylogenetic patterns, epigenetic host-factors and phenotypic plasticity. The *Ty1/Copia *and the *Ty3/Gypsy *families represent the oldest patterns in this network that we found mimics eukaryotic macroevolution. The emergence of the *Bel/Pao, Retroviridae *and *Caulimoviridae *families in this network can be related with distinct inflations of the *Ty3/Gypsy *family, at distinct evolutionary times. This suggests that *Ty3/Gypsy *ancestors diversified much more than their *Ty1/Copia *counterparts, at distinct geological eras. Consistent with the principle of preferential attachment, the connectivities among phenotypic markers, taken as network-represented combinations, are power-law distributed. This evidences an inflationary mode of evolution where the system diversity; 1) expands continuously alternating vertical and gradual processes of phylogenetic divergence with episodes of modular, saltatory and reticulate evolution; 2) is governed by the intrinsic capability of distinct LTR retroelement host-communities to self-organize their phenotypes according to emergent laws characteristic of complex systems.

## Methods

We used distinct *Ty3/Gypsy *and *Retroviridae *LTR retroelements classified at GyDB [26] and diverse protein sequences of known *Ty1/Copia, Bel/Pao *and *Caulimoviridae *elements as queries to search online against the non-redundant database at NCBI [91]. The programs tBLASTn and BLASTp [92] were used for these searches. We collected the outputs involving non-redundant sequences with their available full-length genomes and built a database of 268 non-redundant LTR retroelements. Additional File 1 shows the inferred NJ tree of these sequences providing detailed information about the names, taxonomy, hosts and Genbank accessions of all taxa. Of this set, 28 sequences (summarized in Table 1) correspond to new sequences retrieved from diverse sequencing projects at NCBI.

**Table 1:** New LTR retroelement sequences retrieved from sequencing projects

**Element**	**Group**	**Gene ID**	**Chromosome**	**Host**	**Structure**
*Cubel*	*Bel/Pao*	170058572	Unknown	*C. quinquefasciatus*	LTR-GAG-POL-LTR
*Gabel*	*Bel/Pao*	83921752	Unknown	*G. aculeatus*	LTR-GAG-POL-LTR
*Hydra3-1*	*Bel/Pao*	204801980	Unknown	*H. magnipapillata*	LTR-GAG-POL-LTR
*Kobel*	*Bel/Pao*	154426342	Unknown	*S. kowalevskii*	LTR-GAG-POL-LTR
*Mabel*	*Bel/Pao*	170588120	Unknown	*B. malayi*	GAG-POL
*Nabel*	*Bel/Pao*	156542142	Unknown	*N. vitripennis*	GAG-POL
*Purbel*	*Bel/Pao*	115954126	Unknown	*S purpuratus*	LTR-GAG-POL-LTR
*Spirobel*	*Bel/Pao*	109716041	Unknown	*T. spiralis*	LTR-GAG-POL-LTR
*Tribel*	*Bel/Pao*	86577607	III	*T. castaneum*	LTR-GAG-POL-LTR
*Zebel*	*Bel/Pao*	38304119	VII	*Zebrafish*	LTR-GAG-POL-LTR
*Araco*	*Ty1/Copia*	12321377	I	*A. thaliana*	LTR-GAG-POL-LTR
*Humnum*	*Ty1/Copia*	194680628	Unknown	*H. numata*	LTR-GAG-POL-LTR
*Hydra1-1*	*Ty1/Copia*	194993408	Unknown	*H. magnipapillata*	LTR-GAG-POL-LTR
*Hydra1-2*	*Ty1/Copia*	189515648	II	*Danio rerio*	GAG-POL
*Koco*	*Ty1/Copia*	1255921	Unknown	*D. koepferae*	LTR-GAG-POL-LTR
*Oryco1-1*	*Ty1/Copia*	41223814	XII	*Oryza sativa*	LTR-GAG-POL-LTR
*Oryco1-2*	*Ty1/Copia*	32488601	IV	*Oryza sativa*	LTR-GAG-POL-LTR
*Batata*	*Ty1/Copia*	56407676	Unknown	*Ipomoea batatas*	LTR-GAG-POL-LTR
*Poco*	*Ty1/Copia*	157365037	II	*P. trichocarpa*	LTR-GAG-POL-LTR
*Tricopia*	*Ty1/Copia*	86575464	VIII	*T. castaneum*	LTR-GAG-POL-LTR
*Vitico1-1*	*Ty1/Copia*	123691103	XV	*V. vinifera*	LTR-GAG-POL-LTR
*Vitico1-2*	*Ty1/Copia*	147783960	XVI	*V. vinifera*	LTR-GAG-POL-LTR
*Amn-san*	*Ty3/Gypsy*	111608668	Unknown	*X. tropicalis*	LTR-GAG-POL-LTR
*Amn-ni*	*Ty3/Gypsy*	187466581	X	*D. rerio*	LTR-GAG-POL-LTR
*DRM*	*Ty3/Gypsy*	46848209	IV	*D. rerio*	LTR-GAG-POL-LTR
*Hydra2-1*	*Ty3/Gypsy*	204647056	Unknown	*H. magnipapillata*	LTR-GAG-POL-LTR
SPM	*Ty3/Gypsy*	115913323	Unknown	*S. purpuratus*	LTR-GAG-POL-LTR
DrFV-1	*Retroviridae*	85857417	IV	*D. rerio*	LTR-GAG-POL-AC-LTR

Acronyms are derived from the host name. As an example: Cubel means "Culex quinquefasciatus *Bel/Pao *element". AC means additional or accessory genes.

The most conserved parts (cores) of the PR, RT, RH and INT protein domains encoded by the investigated sequences were used to perform four multiple alignments, one for each domain, using Clustal X 2.09 [93]. Alignments were manually refined using GeneDoc [94] in shade mode assuming the following groups of amino acid similarity: [T, S - small nucleophile amino acids], [K, R, H - basic amino acids], [D, E, N, Q - acidic amino acids and relative amides], and [L, I, V, M, A, G, P, F, Y, W - hydrophobic amino acids]. Next, alignments were concatenated into a single pol alignment using the "Join Alignments" server [95]. For methodological reasons, pol alignment presents the INT domain of all *Ty1/Copia *elements and those of REM1 and *Gmr-1 Ty3/Gypsy *elements after RH, as in the remaining sequences aligned. Using a similar criterion, caulimoviruses are represented with a gap covering the entire INT region in this alignment. Pol alignment is available in various formats within the GyDB collection [96] in the section "Pol" in box "Multiple alignments" at [97].

Pol alignment was used to infer the phylogenetic reconstruction analysis shown in Additional File 1. Subsequently, this alignment was divided into five sub-alignments, one for each retroelement family. We then re-refined each family's alignment using GeneDoc and inferred five phylogenies, shown in Figures 1 and 2. All phylogenetic reconstruction analyses employed MEGA 4.1 [98] and the NJ method with 100 bootstrap replicates under the following conditions: uniform rates among sites and pairwise deletion of gaps. Additionally, we constructed phylogenetic networks based on pol using SplitsTree 4.10 [21]. Details on the methodology used are available in Additional file 2 AF2C.

We investigated diverse phenotypic features such as duplication and variability of amino acid motifs or several gene features that imply different genomic organizations. These were classified as PAMs or as GFs, respectively. We constructed alignments based on the following PAMs: 1) CCHC array at NC; 2) PR pattern 3 motif; 3) PR ILG motif; 4) INT-like GPY/F motif. For the CCHC array, we created a sequence database with all motifs found at the C-terminus of gag. As described in Figure 4, we found five different CCHC array motifs in this database (plus the motif lost), which was subsequently divided in five sub-databases one for each state, these were used as inputs to perform five multiple alignments. For the three other PAMs, we used GeneDoc to select and to split (as alignments) each trait from pol alignment. By position (gaps included), PR pattern 3 lies between positions 60-70 of pol alignment; PR ILG motif between positions 200-203; and GPY/F motif between positions 1568-1574. Next, each PAM alignment was divided into a number of sub-alignments (one for each PAM state as described in Figure 4), which were used as inputs to CheckAlign [99] to construct sequence logos [100]. Conditions used for constructing logos were those previously described in [16,17]. All PAM alignments are available in various formats within the GyDB collection [96], in box "Polymorphic amino acid motifs" at [97]. As GFs we considered the following features: 1) presence/absence or ORF position of four genomic features; 2) INT ORF organization; 3) presence/absence of chromodomain at the C-terminus of INT; 4) *env*; 5) additional genes. Information of these features was retrieved from Genbank accessions or by comparing the sequences with the HMM search at GyDB. Finally, the eight markers were combined in a single MC as follows:



where "*mx*" is one of the eight markers and "*i*" the state of "*mx*".

An exhaustive summary of all markers, their states and the distinct MCs is available in Additional file 3, Sections AF3A.

PAMs' information (except that of the ILG motif) was used to design bipartite networks models capturing network relationships based on single markers under two different scenarios. Bipartite networks are multigraphs in which nodes can be partitioned into two disjoint sets, *P *and *Q*, so that each edge in *E *joins one node in *P *with one node in *Q*. These were constructed following steps 1 and 2 or steps 1 and 3 summarized in Additional file 4 using Mathematica 7.0 [101].

Finally, MCs' information was used to construct an undirected multigraph model to investigate the global network structure of the system, based of the phenotypic proximity of the distinct MCs, in single steps. Undirected multigraphs are graphs in which the same pair of nodes may be linked by more than one edge and where the order of the nodes is not relevant. This model was constructed following steps 1, 4a and 4b summarized in Additional file 4 using Mathematica 7.0 [101]. We calculated the degree distribution *P(k)*, the average clustering coefficient *C*(*k*) and the mean shortest path of this graph using Mathematica 7.0 and the steps 5a and 5b summarized in Additional file 4. The degree distribution is the probability that a node in the network has a degree of value *k *(represented in the horizontal axis). This parameter represents the relative amount of hubs (highly connected nodes) in the network. The clustering coefficient is the proportion of edges connecting a node of degree *k *that form triplets. In short, this parameter is the probability that subsets of distance 2 nodes form highly connected clusters. The mean shortest path is the average of all the shortest distances between pairs of nodes.

## Abbreviations

(AG): Additional or accessory genes; (CA): Capsid; (CCHC): Cysteine zinc finger array; (CH): Chromodomain; (DrFV-1): Danio rerio Foamy Virus Type 1; (ERV-L): Endogenous Retrovirus-Leucine; (GyDB): Gypsy Database; (GF): Gene Feature; (Gya): Giga years ago; (HMM): Hidden Markov model; (ICTV): International Committee on Taxonomy of Viruses; (INT): Integrase; (LTR): Long terminal repeat; (MA): Matrix; (MC): Markers combination; (Mya): Millions of years ago; (MGE): Mobile genetic element; (NC): Nucleocapsid; (NCBI): National Center of Biotechnology Information; (NJ): Neighbor joining; (ORF): Open Reading Frame; (OTU): Operative taxonomic unit; (PAM): Polymorphic amino acid motif; (PR): Protease; (PVCV): Petunia Vein Clearing Virus; (RH): Ribonuclease H; (RT): Reverse transcriptase; (SnRV): Snakehead retrovirus; (YR): Tyrosine recombinase.

## Competing interests

The authors declare that they have no competing interests.

## Authors' contributions

CL and AM conceived and designed the study; CL performed the analyses, characterized the markers, designed the models, and wrote the paper; AMP built the graphs, prepared Additional file 4, and co-wrote the paper; LB performed the phylogenetic reconstruction analyses; HB contributed interesting ideas and views and aged the tree of eukaryotes together with CL.

## Reviewers' comments

### Reviewer's report 1

#### Eugene V. Koonin (National Institutes of Health, Bethesda, MD, USA)

##### Reviewer comments

A comprehensive study on the evolutionary dynamics of LTR retroelements, using several network methods in conjunction with standard, alignment-based phylogenies. I generally agree with the authors that phylogenetic trees are not suited to fully describe the modular evolution of mobile elements such as retroelements, so I think the use of all these network analyses and representations is welcome. The conclusion on the existence of "big bang-like" transitions in the evolution of retroelements (if I understood the authors' meaning rightly) is very appealing. The details on evolution of specific groups of retroelements will be of interest to researchers in the field.

##### Authors' response

We thank this referee for his positive evaluation. Yes, our results suggest inflationary mode of evolution in the evolutionary history of LTR retroelements. This gives support to the idea of that LTR retroelement evolution does not proceeds as a tree but rather a network. The history of LTR retroelements can be traced as network that evolves alternating gradual and vertical means, with diverse episodes of modular, saltational, and reticulate evolution. Our study gives additional support to; 1) the notion arguing that the history of life not as a tree but as a network of networks; 2) the existence of "big bang" patterns in the evolution of biological systems as modeled by this reviewer in prior studies [52,53] (see also our answer to Reviewer 3).

##### Reviewer comments

Despite my overall positive impression of this manuscript, I do perceive several problems at different levels:

1. The repeated and emphatic mention of "adaptive landscape", "functional landscapes", "landscapes of functional strategies" etc in this article does not refer to fitness landscape as a mathematical concept or even a concrete image but rather as a very general metaphor. Along the same lines, the discussion of the power laws and how these apply to evolutionary networks, I think, needs to be more careful and concrete (or just dropped altogether as it adds precious little to our understanding of the evolution of retroelements, or any other class of genetic elements). I think the manuscript would benefit from toning down this rhetoric that has the potential confuse the general audience and to annoy the experts.

2. I do not think that the authors' representation of eukaryotic phylogeny is up to date. In particular, the notion of "crown group" is, I believe, obsolete, and should be abandoned. Neither is it appropriate to speak of a "three-way split" between animals, fungi, and plants. We know for a fact now that animals and fungi are sister groups. The appropriate representation of eukaryotic phylogeny at this time is a star of 5 supergroups (see Keeling et al. The tree of eukaryotes. Trends Ecol Evol. 2005 Dec;20(12):670-6). Actually, the representation of the eukaryotic phylogeny in Figure 4 (very appealing figure, indeed) seems to adhere to the supergroup concept and is unobjectionable to me but the text needs to be brought in sync with this.

3. I think the article would be more complete if the authors at least touched upon the prokaryotic roots of the modules that comprise the eukaryotic retroelements, for instance, the origin of protease of these elements from a distinct family of bacterial aspartic proteases (Krylov DM, Koonin EV. Curr Biol. 2001 Aug 7;11(15):R584-7).

##### Authors' response

1) To avoid excess in both manuscript size and topics, we have removed terms such as "adaptive landscape", "functional landscapes", and "landscapes of functional strategies" from the final manuscript version (see also our response to reviewer 2). However, in our humble opinion the underlying relationship between these terms and the notion of "fitness landscape" merits further attention as it may be not just metaphoric. That is, we noted that the distinct markers and their states evidence variability of diverse functional features of LTR retroelements. This variability gives universality to the network and represents functional phenotypes, which at the molecular level, offer taxonomical differentiation. Note that one can distinguish a retrovirus from a retrotransposon based on the presence or absence of an *env *gene, or can differentiate at least two distinct strategies of integration based on the presence or absence of a chromodomain module at INT [87], etc. It is thus reasonable that, if the features we describe enclose phylogenetically relevant taxonomic states, these suggest variations over function probably related with retroelement speciation. An interesting discussion is if "homoplasy" (convergence) represents a better explanation than "ancestral homology" (ancient divergence) of such universality. The acquisition of GFs seems independent as the original acquisition of such functions is not by inheritance from a common ancestor. This implies that the acquisition of a new mechanism is potentially capable to give selective advantage to the original ancestral lineage that captured the feature. Regarding PAMs, the similarity of their distinct states, the host-dependent hierarchy of these states, and the synchrony among particular states of distinct PAMs, suggest that the diversity of the system expands continuously, within and between families. Taking the *Retroviridae *as an example, one can see that this family can be divided into a number of genera collected in three classes. With a few exceptions, we found that the distinct elements classified in each class show the same markers states. If we now take Class 1 and within it the genus gammaretrovirus, we will find that each gammaretrovirus sequence has a particular marker combination. We originally described this observation as "functional landscape", as the relationship between this and fitness landscape derives from the fact that markers are lineage-specific and can be constituted in phenotypic combinations. Hence, is there is a phenotypic combination that is representative for gammaretroviruses, that is probably because such a combination is the most "adaptive" phenotypic combination for gammaretroviruses. If so, any variation of the usual combination might be representing functional variations in the fitness of a gammaretrovirus, and this can be tested by both empirical and computational methods. On the other hand, the redundancy of marker states observed between certain families is clear evidence of reticulate (network) evolution. This is not trivial regarding the discussion of power laws properties in the diversity distribution of LTR retroelements. Big-bang means can be related with power-law patterns (this is a universal property of real world networks). Taking this into primary consideration, we have rewritten the manuscript and improved several analyses to clarify this context.

2) Done, we removed expressions such as "crown eukaryotes" or "three way split" from the current manuscript version, updated Figure 4 (which in the final manuscript version is Figure 3) and rewritten the related text following the line traced by this reviewer and by reviewer 2.

3) Done, the new manuscript version includes and discusses the clan AA topic at the prokaryotic-eukaryotic transition.

### Reviewer's report 2

#### Eric Bapteste (Dalhousie University, Halifax, Canada)

##### Reviewer comments

The present version of the manuscript requires substantial revisions. It is really difficult to read, both because its lack of focus and the impressive number of typos it contains (too numerous to be listed here!). The English also needs to be significantly improved: the manuscript simply cannot be published as it is. It is a draft. Although there are some interesting ideas in this work (the use of a non-tree-like framework to discuss complex evolutionary scenarios about mobile elements evolution), I do not feel its conclusions are solid enough or at least convincingly presented here.

##### Authors' response

The research has been significantly improved and the manuscript has been carefully re-written to make the language accessible to any reader. We hope that this expert will now find the new manuscript robust and straightforward.

##### Reviewer comments

Certainly, the polyphyly of Retroviridae into the Ty3/Gypsy bio-distribution is clearly showed. It has been extensively studied by the authors through the definition of polymorphic amino acids markers (PAMs), i.e. some key molecular characteristics used to classify mobile elements. Although I agree that Retroviridae origin is likely polyphyletic based on these criteria, I did not find that this fact proved the Three Kings Hypothesis (according to which Retroviridae of classes I, II and III trace to three Ty3/Gypsy sources that emerged at different times during evolution, before the split between Protostomes and Deuterostomes). In particular, I wonder whether the various mobile elements discussed here can not recombine with each other regularly enough, to the point that attempting any temporal polarization of their evolutionary history becomes impossible. To test that, I strongly encourage the authors to perform one more analysis that would value their research. They should use their PAMs to reconstruct a global network including Ty3/Gypsy and Retroviridae elements altogether. Such a network (improving over the fairly abstract figure 3) would display all the connections between these mobile elements and it would thus help deciding whether Retroviridae emerged from relatively well isolated Ty3/Gypsy lineages (or whether this is impossible to tell) and whether these Retroviridae can be suspected of subsequent recombinations (or not). This network reconstruction could be achieved by different means, either through the recoding of the PAMs features in a matrix of characters or by realizing a Splitstree analysis (for instance) of the whole alignments of Ty3/Gypsy and Retroviridae sequences. That way, it would become obvious whether their phylogenetic history is too complex to be told or follows the Three Kings hypothesis. Subsequently, the taxonomical context in which the different elements of this eukaryotic LTR retroelements network are found could be superimposed to it, thus allowing the identification of potential correlations between the taxonomic distribution and the recombination/emergence events in Retroviridae and other LTR retroelements evolution.

##### Authors' response

Done, the new manuscript version includes phylogenetic analyses and (as this expert recommended) network analyses. In particular, we have inferred the evolutionary history of the five groups addressed in the previous study. The phylogenetic inference is an important component of the manuscript. While the evolutionary history of these families has been fully addressed by prior research based on the RT, there is no previous study considering the LTR retroelement system as a whole, based on pol. This was important to elucidate the distinct markers and perform network analyses. With this aim, we used the tool SplitsTree to analyze the evolutionary history of LTR retroelements as a phylogenetic network. Then we superimposed the phylogeny of LTR retroelements over their distributions. The former figure 3 has been removed from this version and its information is redundant over Figures 6 and 7. These two are bipartite multigraphs addressing the same topic but in a more elegant way. From that point on, it is important to clarify that the main objective of the manuscript was not to test the three kings hypothesis but to provide new insights on the network mechanisms ruling in the evolutionary history of LTR retroelements. In this issue, the three kings hypothesis is a set of starting conditions to activated our attention to the possibility of a network history. In fact, while in the present paper we have tested such a hypothesis in diverse manners following the reviewer's indications, it was addressed and published in a prior paper [16]. On the other hand, we assumed various mechanisms of evolution including recombination but we did not try to test them. This is because their existence is well supported by prior publications investigating recent evolutionary patterns. We two interesting examples of recombinant histories in the potential *Athila *retroviruses of plants [9] and the HIV-like *Retroviridae *retroviruses [10] of mammals, whereby new forms emerge from the recombination of subtypes. Most ancient cases are difficult to test probably because LTR retroelements can recombine with each other regularly enough, to the point that attempting any ancient temporal polarization of their evolutionary history becomes a daunting task. Testing reticulate events between distant counterparts is extremely difficult because of 1) the different rates of evolution of the distinct genomic regions of the retroelement taxa; 2) the wide divergence between sequences accumulated during evolution; 3) extrinsic constraints; 4) conflicting signals, etc. For instance, based on the RT and RH the *Retroviridae *are more similar to each other than to any *Ty3/Gypsy *lineage but the PR and the gag polyprotein show different perspectives.

##### Reviewer comments

The authors could also discuss whether the variation of their Diversity Index H may be biased and partly due to an unequal taxonomical samplings between Deuterostomes, Protostomes, Viridiplantae and Fungi's mobile elements. In particular, they could establish whether the increase in diversity of mobile elements in Opisthokonts is significant. Presently however, the proposition that "many of these events [at least three independent radiations of mobile elements] are coincident with the major biological transitions and changes in molecular and cellular complexity of eukaryotes ", although seducing, does not seem fully justified by the material and analyses presented here. (In my view, this same criticism also holds true for the presumed "ancient radiation of mobile genetic elements that is as old as the transition from prokaryotes to eukaryotes" evoked - yet not tested- by the authors). To summarize, to justify that the big bang model better fits the data than the tree model, the manuscript should be more focused on Ty3/Gypsy and Retroviridae evolution and should better value the PAMs description and their analysis.

##### Authors' response

The diversity Index H analysis has been removed and substituted by network and relative frequency analyses. In particular, the notion of inflationary means of evolution is shown in figures 4-9. On the other hand, the set of sequences - 268 - used in this study is non-redundant and covers the most representative lineages of each family in plants, fungi, animals, and unicellular organisms. There is no bias in this study because it includes the non-redundant diversity of LTR retroelements to date known as well as a number of new sequences introduced in this study (summarized in Table 1). This means that we not only focus on all known lineages of LTR retroelements but also on new sequences and lineages addressed in this study (see, Figures 1 and 2). The three biological transitions were calibrated according to the most likely age of the LTR retroelement host as phyla. This has been conducted taking into primary consideration previous studies with a focus on molecular estimations and the fossil record. The transitions cover: 1) from the earlier eubacterial fossils and the first traces of unicellular algae eukaryotes until the segregation of crown eukaryotes into plants, fungi and animals: 2) From the split of plants, animals and fungi to the Cambrian explosion, the rise of vertebrates, the emergence of land plants, etc; 3) the origin of the gymnosperms; the split of amniotes into reptiles and mammals, the massive radiation of winged insects; and the emergence of flowering plants. The perspective of an ancient radiation of LTR retroelements is supported by the wide distributions of all LTR retroelement families and their differential distribution in eukaryotes. *Ty3/Gypsy *chromoviruses and the *Ty1/Copia *family are both widely distributed in the genomes of not only red and green algae but also in plants, fungi and animals. This derives in the common assumption these two are the most ancient LTR retroelement patterns in eukaryotes (see [7,63]). A similar criterion applies in the relationship between distribution and age of the remaining LTR retroelement families. We think that figure 4 is the test asked by this referee, as it is a graphical description of what the post-genomic era suggests at this point over this topic.

##### Reviewer comments

Editorial changes to be considered:

The background section is very complex (with lots of information) and it is simply impossible to understand (for a non specialist) without a figure. This section needs extensive rewriting to clarify how the PAMs are defined and their nature. Interestingly, such a figure almost exists in the manuscript - it is figure 1-, and it should be introduced much earlier than page 8 (if possible by mapping on it the classes I, II, and III).

##### Authors' response

The whole manuscript has been completely rewritten and restructured. It includes a background section more accessible to any reader in broad terms. Much of the information mentioned by the expert has conveniently been moved to corresponding subsections under 'Results and discussion' and complemented by an improved version of Figure 1, although now it is Figure 3. The current manuscript is conducted appropriately to reach and understand the background of this figure.

#### Reviewer's comment to the revised manuscript

##### Reviewer comments

The paper by Llorens et al. entitled "Mapping the landscaping network principle in explaining the diversity and evolutionary patterns of eukaryotic LTR retroelements" is both too long and too complex (and in places hard to understand). It requires significant revisions. This manuscript should be:

1. shortened

2. clarified

To focus on its main original point, the proposition of a network framework to analyze the evolution of eukaryotic LTR retroelements and to show that, once a "good" combination of characters is obtained, then the LTR element mostly evolve within a lineage.

##### Authors' response

The final manuscript has been improved and reduced in size (we have removed 13 pages of the last version) to make it shorter and clearer than the former version as suggested by this reviewer.

##### Reviewer comments

To this end;

a) all the section entitled " Phylogenetic patterns of LTR retroelements based on pol" (p.3-11) could be significantly shortened (eventually removed or introduced as Supp. Mat). According to the authors, this part does not provide any really new perspective on the issue (cf. p.11), and truly the core argument of their paper does not start before p. 11 anyway

b) the text from "Under this scenario, " (p. 12) to 'in turn evolving in a tree-like fashion" (p.13), if summarizing ref. 19 can be removed.

c) almost all their figures, but figures 4 and 5, can be removed. Most of the networks presented here are too complex, in particular those with two kinds of nodes. It is not pedagogical. What the authors should do is reconstructing networks with one type of nodes (for instance the taxa) and connect them when two nodes (two taxa) share a common property (out of the 8 characteristics listed by them to define an LTR element). Alternatively, they could connect such nodes when they share a given combination of characteristics. This could be the only figure of their paper, and it would make their point. If they want to provide more in depth information, they could color the edges and the nodes of this single network according to various criteria (taxonomy, number of copies in taxa...) thus mapping any additional information they think is relevant.

d) there are many sentences, starting by the title, that are impossible to understand and should be rewritten. Sentences like:

- "Here new strategies emerged passively and overlapped with prior strategies until configuring a complex network, whereby retroelement lineages co-opted the most adaptive landscape, depending on their molecular dynamics and host distribution. " (p.2)

- "Here, it is important to emphasize that the three kings hypothesis does not intend to establish a direct relation between these lineages but that argues three *Ty3/Gypsy *ancestors in the evolutionary history of the *Retroviridae *common ancient poly- or paraphyletic scenario of diversity yet to be understood. » (p. 18)

- "At the LTR retroelement level, the functional landscape of a LTR retroelement taxon can vary, or be more or less successful, if it gains or looses a marker or if a marker in this taxon evolves from one state into another." (p.24)

- "Thus, new strategies emerged passively in the LTR retroelement system and overlapped prior strategies until configuring a network, whereby retroelement lineages co-opted the most adaptive landscape, depending on their molecular dynamics and host distribution. That is "the landscaping network principle" in explaining the diversity and evolutionary patterns of eukaryotic LTR retroelements. » (p.26) should be clarified or removed from the paper.

e) The notion of "landscape" is confusing and poorly explained. The analogy may fit or it may not. But the use of the word deserves more careful, direct, efficient explanations. The same criticism applies for the notion of "functional strategies". What does it mean? This lack of clarity entails that the notion of "landscaping network principle" is almost impossible to understand.

##### Authors' response

a) Phylogenetic analyses were performed in order to follow the line traced by reviewer 3. It would be conflictive if we remove such information. We have however reduced the text as much as possible, according to the criticisms of this reviewer, and particular care was taken in the process. In addition, it is important to highlight that the perspective that remains unchanged since 1998 (page 11) is the knowledge over the deep evolutionary history (the ancestral evolutionary relationships among families) and that phylogenetic reconstruction analyses cannot convincingly resolve this part of the LTR retroelement history. Actually, this is the foundational topic in this manuscript. Phylogenetic inference is essential as it is the most robust method to classify the OTU diversity into families and lineages. Our study makes an important update over prior knowledge of LTR retroelement diversity (and taxonomy). This is covered in the section entitled "Phylogenetic patterns of LTR retroelements based on pol", where we investigate the diversity patterns of all families and provide diverse and previously unpublished results.

b) Done in the manuscript.

c) Indeed, there are many methods to construct network models. Those we applied are well documented in graph theory, the area of mathematics that deals with the mathematical foundations of networks. Bipartite multigraphs (those with two types of nodes) are not complex; in fact, one of their properties is their simplicity. What is complex (because of its diversity) is the analyzed LTR retroelement system. Bipartite multigraphs are appropriate to evaluate the history of LTR retroelements based on two or more independent features, as this history does not depend only on a single feature. In this case, we considered markers (node 1), host distributions (node 2), and taxa or lineages (links). The obtained results can be quantitatively and qualitatively interpreted because the density of each set of links gives a clear vision of the frequency distribution of each PAM state under two independent scenarios. However, to simplify the final manuscript version (according to the general comment of this reviewer, "the manuscript is too long and too complex") bipartite multigraphs have been removed from results and are now provided within Additional file 3. On the other hand, we tested various graph methods. Constructing networks using the distinct taxa as nodes and the distinct states of a single maker as links only offers information regarding such a marker. By itself this kind of model is not very informative unless it integrates phylogenetic information. Note, for instance, that should we join "skipper" with "maggy" and "CoDi 7.1", based on a CCHC marker state, we have nothing in particular except that these sequences share that feature. For this reason it is important to resolve the multiple phylogenetic patterns. Constructing networks using taxa as nodes and using all markers as edges gives an impressive number of edges among all nodes. This results in a complete graph because the distinct markers have states, which are common not to two retroelements but to a number of them. Moreover, a systemic shortcoming with this model is that, in most cases, multiple distinct links among sequences within lineages mask other relationships which are a priori more interesting (e.g. those based on distinct families). In contrast, we found markers of reticulate evolution, which allowed us to overcome the mentioned obstacle. The most interesting aspect of these markers is their universality. Using combinations of markers we have phenotypes associated to phylogenetic identities. This allows a better interpretation of the network evolutionary dynamics, etc. Taking this into primary consideration and recognizing the contribution of this reviewer to the paper, we agree with him that the work needs a more explicative network framework addressing the main original point. This point is close to the feeling of this expert as noted in his phrase; "the proposition of a network framework to analyze the evolution of eukaryotic LTR retroelements and to show that, once a "good" combination of characters is obtained, then the LTR element mostly evolve within a lineage". With this aim we have rewritten the manuscript and revised, improved, and adapted undirected graphs to make them more comprehensible in the line traced by this reviewer.

d) The sentences addressed have been revised and restructured in order to better convey their intended meaning.

e) We have changed the term "functional landscape" by other terms such as "marker combination" and/or "phenotypic combination" (see also our response to Reviewer 1, in regard to a similar question). For the same reason, we have changed the title of the manuscript to a more appropriate one.

##### Reviewer minor comments

The authors should be more careful in interpreting "absence" if sequences from incomplete genomes are considered in their analyses. The possibility of an undersampling of the LTR retroelements should be discussed.

##### Authors' response

The study contemplates all currently known *Caulimoviridae *and *Retroviridae *genera considered at ICTV, plus a new spumaretrovirus sequence we introduce for the first time in this article. We also consider a number of *Ty3/Gypsy, Ty1/Copia *and *Bel/Pao *LTR retroelements. This not only covers all commonly known lineages but also extends knowledge on the phylogenetic diversity of these families (we describe new sequences and new lineages). Another question is if such a sample is a good approximation of the true diversity of LTR retroelements in eukaryotes. Upon this, the study evaluates a number ofnon-redundant *Retroviridae *retroviruses ranging from distinct fishes, amphibians and sauropsids to mammals. We equally consider a number of *Ty3/Gypsy, Ty1/Copia *and *Bel/Pao *LTR retroelements retrieved from the genomes of distinct protists, plants, algae, fungi, amoebas, plathyelminthes, nematodes, cnidarians, crustaceans, winged and non-winged insects, echinoderms, urochordates and vertebrates. We believe that this gives sufficient information to extrapolate conclusions and perspectives over both LTR retroelement evolution and macroevolution, even if some of the used host genomes are incomplete to date. We have shown that plants have particular lineages of LTR retroelements, and so do fungi, protostomes and deuterostomes, etc. Indeed, if one were to investigate LTR retroelements in the genome of a new flowering plant, a priori anything might be found, but what any expert in the topic expects to find are chromoviruses, *Athila/Tat Ty3/Gypsy *elements and *Ty1/Copia *elements. That is, it is really difficult to think that one can find a *Retroviridae *or a *Bel/Pao *sequence in such genome. However, should such a rare case happen, the most probable explanation is first, lab contamination, and second, a very rare horizontal transfer, an exciting exception meriting an important publication (despite all, nothing is impossible). With this manuscript we update prior perspectives but we are sure that further availability of data will help to improve and calibrate the introduced framework. With this, our conclusions are not problematic or biased are just a point in which we recapitulate prior knowledge, contrast current information, and offer a new framework for further evaluation (see also our response to reviewer 3, in regards of the same question). Another question is the presence of evolutionary gaps due to the loss of lineages associated to eukaryotic extinctions. Such a bias is plausible but we think that it is not an inquisitive obstruction for studying and modeling the evolution of LTR retroelements or that of their hosts. In fact, this is another important argument supporting the idea of using markers combinations instead of taxa. Networks based on combinations are not biased by the sampling or by the number of sequences used for each lineage. Even more interestingly, they give some clues about putative extinguished forms or extant uncharacterized ones, as we have illustrated in current Figure 5.

##### Reviewer minor comments

The authors indicate that their pol data contained multiple distinct splits, and that these splits can be explained by different modular, reticulate, and saltational evolutionary events (p. 11). They could also be due to "mutational saturation", which should be discussed.

##### Authors' response

We cannot dismiss mutational saturation in diverse traits of a retroelement genome. However, the sequences used in this study correspond to coding sequences from which we extrapolated their most conserved parts (cores) to perform alignments. All these cores show lineage specific phylogenetic signal. That is, all sequences of all lineages are more similar to each other than to other sequences, or in other words, we did not observe multiple substitutions in these traits that could lead us to think that their signal is random (mutational saturation). However, the discussion of this possibility is important and has been addressed in results to make an emphasis in the distribution of PAMs in both phylogeny and host distribution, which cannot be explained by random patterns.

##### Reviewer minor comments

The authors should use a better phylogeny of reference (see Simpson and Roger in Curr. Biol.): their knowledge of the protist taxonomy is a bit too vague (see for instance strange notions such as the supposed "three way split of plants, animals and fungi" (p.15), the odd branching of diatoms that the authors said are "informally classified as protists" (p. 18)...)

##### Authors' response

Done. For simplicity's sake we used reference [41] suggested by Reviewer 1 (see the comments of reviewer 1), as it is compatible and a bit more recent than the one suggested by this reviewer. In a similar manner, the expression "three way split" has been removed from the manuscript. Regarding diatoms, as far as we know, they are considered as chromalveolates. The root of chromalveolates in former Figure 4 (now Figure 3) delineates a star tree together with the remaining supergroups. That is correct on the basis of the investigated hosts and their LTR retroelement sequences we classify. Bear in mind that our study focuses on the evolutionary history of LTR retroelements, not on the most accurate tree of life topology.

##### Reviewer minor comments

Likewise what are "crown" eukaryotes (p. 15)? Or rather what eukaryotes are not crown eukaryotes? This notion is problematic. And so his the notion of reptiles (a grade not a clade) for a phylogenetic-based interpretation (p.17). Strictly speaking, sentence like "In tetrapods, for instance, the *Ty3/Gypsy *and the *Retroviridae *distributions overlap until reptiles, but there is no evidence of functional *Ty3/Gypsy *elements in other amniotes (neither *Ty1/Copia *nor *Bel/Pao*). " (p.17) are problematic (and meaningless) for many biologists trained as cladists. (This can be of concern since the authors seem to embrace the cladistic logic when they reject protists since these taxa are a paraphyletic group, p. 18).

##### Authors' response

The manuscript has been amended to offer a commonly accepted vision. In particular, we removed the terms "tetrapods" and reptiles from the text and use terms such as sauropsids or synapsids. In regard to protists we do not reject them, we just noted that as a group they are not considered monophyletic. We have however adapted Figure 3 according to the most recent trends over the tree of life. We hope this referee will find the new topology appropriate.

##### Reviewer minor comments

Are the first trace of unicellular algae really as ancient as 3,500 Mya? (p.14)

##### Authors' response

The text did not exactly claim this. What we said is that the first transition covers "from the earlier eubacterial fossils and the first traces of unicellular algae eukaryotes to the segregation of crown eukaryotes into plants, fungi and animals" (this is from 3,500 to 1500-1330 Mya). We have rewritten the text to clarify this and have changed "crown eukaryotes" by a more appropriate term.

##### Reviewer minor comments

Some claims should be slightly toned down, such as "The distribution of *Ty3/Gypsy *chromoviruses not only in algae [21,68,69] but also in land plants, amoeba [32], fungi and animals [20,21] indicate that these constitute the oldest branch of *Ty3/Gypsy *LTR retrotransposons." (p.14) It does not "indicate" this, it "suggests" it at best. What if these elements were moving over large taxonomical distances? Broad distribution, in presence of lateral transfers, is hard to interpret as ancient common ancestry. The same comment applies to the sentence "It is thus unclear which of the two represents the oldest phylogenetic pattern in the *Ty1/Copia *family, but the wide distribution of both branches indicates that the *Ty1/Copia *family co-existed with chromoviruses before the segregation of the crown group into plants, fungi and animals (1,550 Mya according to molecular dates [71,72])" (p.14), and p.19.

##### Authors' response

Done, the above points have been rewritten with the required care, and following the reviewer's recommendation. However, it is important to emphasize that, when evaluating LTR retroelements, broad distribution in presence of lateral processes is not hard to interpret, as these lateral processes happen between organisms of the same phylum. Caulimoviruses infect plants, retroviruses of insects normally infect insects, those of vertebrates infect vertebrates, etc. This means that there are biological barriers imposed to caulimoviruses and retroviruses and that these barriers have an evolutionary meaning. On the other hand, it is obvious that while there are some examples of LTR retrotransposons believed to be horizontally transmitted, the usual means of LTR retrotransposon transmission are germ lines. This is commonly accepted and indicates that the wide distribution of the LTR retroelement diversity is a sign of deep ancestry. This is so even if we characterize events of lateral transfer, which usually only occur among closely related biological species. Detecting ancient events of lateral transfer is a daunting task but in certain cases can be mapped with reservations (note the discussed example of chromoviruses in fungi and vertebrates addressed in this paper). In one way or another, this suggests that LTR retroelements can be used as evolutionary markers of their host evolution.

##### Reviewer minor comments

I was unable to visualize the figures of network in the supp. mat.

##### Authors' response

As indicated in methods, these files are not figures. Such material is provided as Mathematica notebooks, so that any other author can reproduce our analyses using the tool "Mathematica". To facilitate visualization and management of this material, the final manuscript version joins Table 2 and all these notebooks in a mini web-site provided as Additional file 4 (for more details, see Methods).

##### Reviewer minor comments

There are additional sentence for which the meaning is unclear:

"These markers indicate that the diversity patterns of LTR retroelements are not casually distributed. » p. 2 What does casually means?

##### Authors' response

The intended word is "randomly", changed in the manuscript.

##### Reviewer minor comments

"The model finds support in the phylogeny of LTR retroelements superimposed over their distributions." What distributions? Taxonomical ones?

##### Authors' response

Distributions refer the distinct host distributions of LTR retroelements. We have clarified in the manuscript.

##### Reviewer minor comments

"The evolutionary history of LTR retroelements is not a tree but a networking system evolving in a tree-like fashion" (p.11) is a bit confusing. It should be improved somehow. Same thing for "Under the assumption that functional landscapes have not evolutionary meaning, what we would expect is no redundancy among them." (p.23) and "As this number is much closer to the number of LTR retroelement lineages elucidated than to the number of LTR retroelement taxa we can conclude that the landscapes are lineage specific equally to the markers constituting it. " (p. 23)

##### Authors' response

Again, these points have been rewritten. We hope that the revised text is now appropriately exposed.

##### Reviewer minor comments

The English is much improved (there are still a few oddities:"fossil register" should be "fossil record"

##### Authors' response

Changed in the manuscript

##### Reviewer minor comments

How can biological transitions be edges on the multigraph network? (p.31)

##### Authors' response

Transitions constitute links; it is a relationship derived from the distinct LTR retroelement hosts. Anyway, to clarify understanding of the manuscript, we substituted the model based on transitions by a more appropriate model more according to previous points addressed by this reviewer.

### Reviewer's report 3

#### Emmanuelle Lerat (Université de Lyon, Villeurbanne, France)

##### Reviewer comments

In this paper, Lloréns and Moya, using the occurrence of protein markers in different products of retroelement and retroviridae sequences, have matched the combination of signatures according to the host species in order to determine the link between elements. This approach is by some extend similar to other methods consisting of phylogenetic reconstructions based on presence/absence of genes. The approach is quite interesting and gives a different view of the classical phylogenetic representations.

##### Authors' response

We thank this expert for her help and positive feedback on the background's manuscript. This new manuscript version is a great improvement over the former version. It is co-authored with three other researchers and includes more sequences of not only the *Ty3/Gypsy *and *Retroviridae *families but also of the *Bel/Pao, Ty1/Copia*, and *Caulimoviridae *families. Based on this material we have performed more and new analyses following the line traced by this and the two other referees. We hope this referee will find this new manuscript version improved and useful.

##### Reviewer comments

I have however some criticisms upon different points and speculations made in this paper. The first problem can seem trivial but I am really concerned by the fact that all the article is based on another manuscript by the same authors currently under submission in another journal. It is completely unusual. Generally journals do not allow reference to any publication unless it is at least accepted. What if the other manuscript is never accepted? What if reviewers point particular problems that would completely change the conclusions concerning the existence of the protein markers? That would be a complete paradox. I don't know what is the position of Biology Direct upon such a problem, and in doubt I will assume the other submitted paper as accepted even if it is absolutely not satisfactory for me.

##### Authors' response

In this regard, we think that the policy of Biology Direct is similar to that of other journals (on the basis of our experience with this journal). Our aim when submitting the first version of this manuscript to Biology Direct was just in order to have it reviewed in advance, but we were aware that we should/must wait regarding this publication until having the first manuscript published (currently available under the following citation [16]).

##### Reviewer comments

In the introduction, the authors present the different families of *Retroviridae (*alpha, beta etc.), and the different classes I, II, III. I think that a clearer explanation about the relationship between the two classifications and also where the Ty3/gypsy are positioned in this system would greatly help readers that are not familiar with such classification. About classification, a reminder of what contains the Ty3/gypsy group seems essential (with a table for example) and would facilitate the comprehension of some parts in the paper. For example, p5 in introduction, it is said that GANG architecture of class I is similar to several Ty3/gypsy and that GIGG of class II is similar to Ty3/gypsy lineages like micropia/mdg3 and others. That means that we find the signature of the two classes in different members of Ty3/gypsy. Maybe you should use the term metaviridae to name the global group Ty3/gypsy to avoid confusion? I would also point out that chromoviruses are classified as members of the Ty3/gypsy group (Gorinsek et al. 2004). The references of "phyla", "sample", "lineage", "clade" are also confusing. The authors should homogenize the paper on that point.

##### Authors' response

The text in the section "Background" referred by this reviewer has been moved to "Results" where we make a description of all used evolutionary markers. The new manuscript version presents new phylogenetic and network analyses. In particular, we performed a comprehensive phylogenetic analysis of all families based on the pol polyprotein (see methods). In this new version, we deal with a non-redundant set of 268 sequences belonging to the five aforementioned families, many of which are introduced for the first time in this manuscript (see Table 1). This new manuscript version also presents and discusses a phylogeny for each family and other based on all LTR retroelements (provided in Additional file 1). This will help the readers have a perspective on the multiple distinct phylogenetic patterns in the LTR retroelement system, which in turn helps in clarifying why the different markers are lineage-specific within families or redundant among families. This is the network basis, which as this referee indicates means that we find two or more states of the same signature in different lineages of the *Ty3/Gypsy *family and all other investigated families. Regarding chromoviruses, in the previous manuscript version we did not intend to separate this branch from the *Ty3/Gypsy *family. We apologize if such a perspective was implied in any way. In fact, previous to the approach of Gorinsek et al. cited by the referee and the corresponding author, together with other researcher (Dr. Marin), described chromoviruses as *Metaviridae Ty3/Gypsy *LTR retrotransposons [28]. Before, Wright et al. had originally described chromoviruses as a *Ty3/Gypsy *class [79] and later, shortly before Marin and Llorens work, Malik and Eickbush described the chromodomain at INT of these *Ty3/Gypsy *LTR retrotransposons [4]. The contribution of Gorinsek and Kordis et al. in [63] was important and overdue as they showed (among other pieces of evidence) that chromoviruses are the most ancient branch of *Ty3/Gypsy *LTR retroelements (an important evolutionary perspective supporting this paper). Anyway, we have rewritten the manuscript to clarify that chromoviruses are *Ty3/Gypsy *LTR retroelements. It is difficult however to refer chromoviruses simply as *Metaviridae *elements because, as we show, they constitute the largest phylogenetic branch in the *Ty3/Gypsy *family, while the remaining *Ty3/Gypsy *retroviruses and LTR retrotransposons (including the *Errantiviridae*) fall in another branch. Of course, chromoviruses are *Metaviridae Ty3/Gypsy *LTR retrotransposons, but perhaps what the current LTR retroelement classification needs is more taxonomical levels such as groups, orders, families, classes, genus, clades, etc. The establishment of these levels is a daunting task because they should be concomitant with 1) the phylogenetic patterns of each family; 2) the diversity patterns common to all families, which in turn are polyphyletic. We think that the network shown in this paper may help in this regard. However, meanwhile the current *Metaviridae *classification is discussed, we would appreciate if this kind reviewer would allow us to simply describe chromoviruses as *Ty3/Gypsy *LTR retrotransposons in order to avoid higher confusions (note for instance that the *Errantiviridae *are *Ty3/Gypsy *elements, but they are not *Metaviridae *elements). Finally, the three classes pointed by the reviewer are specific in the classification of *Retroviridae *retroviruses. This means that no *Ty3/Gypsy *lineage has place (to date) in such a classification (see [16,35] and references therein). As suggested by this reviewer, we have rewritten the manuscript to avoid confusion within and between LTR retroelement families and between these and the eukaryotic taxonomies. We use the terms "genus" and "class" when referring to retroviruses and viruses such as *Retroviridae *and *Caulimoviridae *according to ICTV and most recent taxonomical approaches (see [16,35] and references therein); we use the terms "genus" and "clade" to describe the *Ty3/Gypsy*, the *Bel/Pao *and the *Ty1/Copia *lineages supported by bootstrap, which are in agreement to current ICTV classification or are commonly accepted in the field; and use the term "branch" for describing (with just descriptive purposes) the deep clustering elucidated in this study regarding the *Ty1/Copia*, the *Ty3/Gypsy*, and the *Bel/Pao *LTR retroelements. We hope this expert will find now the topic cleaner and clearer for any reader and the manuscript straightforward.

##### Reviewer comments

The material and methods part is not clear. You don't need to present all the content of the GyDB database as there is already a publication on it. It would be better to know exactly what element you have analyzed from which species.

##### Authors' response

Done, we have re-written the whole manuscript, but we took particular care in to remove all references to the GyDB except when it is strictly necessary (citation, URLs, etc). The new manuscript version includes an inferred phylogeny of all sequences used (Additional file 1) with information of the names, hosts, and Genbank accessions of all sequences. The file is provided in html format so any reader can directly access more information and/or the Genbank accession of each sequence by clicking its name or acronym in Additional file 1.

##### Reviewer comments

The authors propose to compute an index variability for each element based on the different possible combinations of signatures. The results are shown on table 1. It is probably an interesting way to have a rapid look at the diversity present in species. The trouble is that the authors never really refer to this table and they don't either explain what they are expecting from the values. Especially how the H value is supposed to vary? I don't really see the point of computing this index as it is almost not interpreted and used in the paper.

##### Authors' response

In the new manuscript version of this work, the index H is redundant over the set of different network analyses performed and was removed from this particular issue. See also our response to Reviewer 2. In particular, network analyses shown in Additional file, 3B-C present similar perspectives than the previous index H but in turn they are more informative and presented in a more elegant way.

##### Reviewer comments

A particular assumption is made concerning the observations of the authors and a theory emitted by E. Koonin concerning evolution. I think this is a clear over statement. First of all, the observations made by the authors cannot be taken as proof that each increase in diversity is coincident with major biological transitions (as stated p22 in discussion). There are no time scales and too many missing data concerning the representation of species and elements to allow such conclusion. Concerning the adequacy with the theory of Koonin, this is also quite speculative. Again, the scales are not the same. Transposable elements and viruses are known to be able to quickly evolve by gene acquisition and genome recombination between elements. When a new element is formed it does not mean that a new host species is born. Even if in some case, the link between mobile elements and speciation is possible, I encourage the authors to be more prudent with their conclusions.

##### Authors' response

Done, we toned down the speculation and refer only the Koonin's model where it is strictly necessary. It would be interesting to highlight that while the scales are different we describe a similar evolutionary means at the molecular level. The evolutionary principle is invariant and while several points of the Koonin's model might be debated (for instance, are the evolutionary phases of Koonin's model really fast and lower steps or they are simply transitions?) our study gives support to the notion of an inflationary mode of evolution. This is exactly the perspective derived from Figure 3, which is an evolutionary map based on the diversity and distribution of LTR retroelements in eukaryotes. We therefore think that our study gives support to the commonly assumed notion that viruses and mobile genetic elements are evolutionary indicators of the evolutionary history of their hosts and vice versa. In the previous manuscript, our interpretation when relating the evolutionary history of LTR retroelement with that of eukaryotes was in order to point the role of mobile genetic elements as evolutionary vectors in the evolution of eukaryotes towards the complexity (as suggested in [1] and other studies), not that the origin of a new LTR retroelement lineage will raise to the born of a new biological species. To clarify this whole framework we have carefully rewritten the manuscript, where we have toned down the speculation and aged the different LTR retroelement host distributions, according to prior molecular estimations and data derived from the fossil record. We hope this expert will find now this scenario better presented and supported by previous research.

##### Reviewer comments

The authors propose the hypothesis of the 3 kings as originators of *Retroviridae *and *Ty3/gypsy *elements. But they temper their position in the discussion p26 saying that there could be more than three classes. They also propose that adding the endogenous retroviruses could possibly change this view. The thing I don't understand is why not having added endogenous retroviruses in this analysis. I don't think that a particular analysis dedicated to these elements makes sense. Moreover, I am wondering if the observed diversity mainly in vertebrates concerning *Retroviridae *is not biased by different effects like a bias in species sequencing. It seems that the high diversity observed in deuterostomia is mainly due to the retroviruses. I am not convinced that there are less diversity in arthropods or even in plants. You can also imagine that other viruses have been more successful in plants for example that are not represented here because they are not member of the retrovirus classes.

##### Authors' response

The main focus of this research is the network history of not only the *Ty3/Gypsy *and *Retroviridae *families but also in all other LTR retroelements and caulimoviruses. Again, we apologize for the way we prepared the first manuscript version, where the *Ty3/Gypsy *and *Retroviridae *families seemed the main focus of research. The *Ty3/Gypsy *and *Retroviridae *network and the three kings hypothesis were published in [16]. In this paper these are the starting conditions that activated the study. To clarify this, we have entitled the manuscript with a more appropriate title. Regarding the question addressed by this reviewer, the new manuscript version collects both "Results and Discussion" in a single section. Here, we test the three kings hypothesis in diverse manners (see also our answer to Referee 2) but the main manuscript focus is the network principle. In fact, the most precise way to investigate the *Ty3/Gypsy *and *Retroviridae *network is by studying it based on all LTR retroelement families known to date. On the other hand, the three kings hypothesis does not argue that all *Ty3/Gypsy *and *Retroviridae *LTR retroelements evolve from three common ancestors but that the three *Retroviridae *classes delineate *Ty3/Gypsy *ancestors in the evolutionary history of *Retroviridae *retroviruses. This sounds similar but it is not the same. The three kings hypothesis neither proposes that Class 1 directly evolves from *Athila/Tat *elements, nor Classes 2 and 3 evolve from *Micropia/Mdg3 *clade and errantiviruses, but that all of them evolve from a common ancient poly- or paraphyletic scenario with different times of emergence. Our study (in both versions) includes a number of endogenous *Retroviridae *retroviruses. In particular, the two *Retroviridae *genera - gammaretrovirus and betaretrovirus - are rich in both endogenous and exogenous retroviruses (for more information in this topic see also [35]), so we certainly implement a wide number of *Retroviridae *endogenous retroviruses in our study. There is not a significant bias for the different *Retroviridae *species used. This can be seen in Additional file 1 and in Figure 3, which show how the multiple distinct *Retroviridae *sequences we evaluate cover a wide host range, from fishes, amphibians, reptiles and mammals. The range covered by this framework has sufficient information to perform hypotheses and conclusions because this is what the post-genomic era suggests at this point. As this reviewer has interestingly pointed out, the different gaps in this biological history, which are derived from bias in the availability of sequenced genomes, will calibrate this map in the future. However, this is not a bias inherent in the corpus of available data. Our study is a first step of current and further research value, and in agreement with previous arguments [38], "surveillance for emerging diseases should extend to sampling and characterization of the entire panoply of viruses, which are circulating not only in people but also in animals" (and all other organisms). Obviously, we temper our position regarding the three kings hypothesis because further sequencing projects might reveal in the future that the diversity of *Retroviridae *retroviruses needs more genera and taxonomical classes to be explained. However, if this occurs, it will not be due to a biased hypothesis, but due to an update based on more and better information. This will not change the fact that while Ty1/Copia, *Bel/Pao*, and *Ty3/Gypsy *LTR retroelements spread in both protostomes and deuterostomes, the *Retroviridae *are only distributed among diverse deuterostomes. This by itself shows that the diversity of LTR retroelements in deuterostomes is greater than that of protostomes and plants, even if we include caulimoviruses of plants (as we have done) in the study. The known to date genomes of plants and fungi, show only *Ty3/Gypsy *and *Ty1/Copia *elements and it is difficult to think that further sequencing data will change this scenario. Moreover, this study includes caulimoviruses because of their retroelement-like gag-pol component close in similarity to the *Ty3/Gypsy *family. While caulimoviruses stay in a separate system of classification because they are DNA viruses, their diversity based on evolutionary markers in their gag-pol component is lower than that of *Retroviridae *retroviruses. At this point, it is important to highlight that this study focuses only on LTR retroelement s (we included caulimoviruses because they evolve from this system). However, we do not known if plants, fungi, protostomes and deuterostomes are more or less diverse in other types of RNA or DNA viruses and mobile genetic elements because the topic addressed in this study is only the particular system of LTR retroelements and related caulimoviruses.

#### Reviewer's comment to the revised manuscript

I have read the new manuscript and the responses made by the authors. They have indeed made a lot of modifications that make the manuscript very interesting and much more clear than the first version. I don't see any new comments and I think the article can be published.

## Supplementary Material

Additional file 1 **LTR retroelement phylogeny**. Inferred based on pol using the 268 LTR retroelements used in this study. This tree includes information about names, Genbank accessions and hosts of all LTR retroelement taxa used. By clicking on each OTU in this tree, the user can download a GyDB file or Genbank accession of the requested element from GyDB or NCBI, respectively. Click here for file

Additional file 2 **Comparative analyses**. Presented through an Excel file divided into four sections; AF2A) C-terminal module of CoDi-A-like Ty1/Copia INTs with significant similarity the chromodomain consensus; AF2B) hits of similarity using different core sequences of DrFV-1 as queries against HMM searches at GyDB; AF2C) phylogenetic networks; AF2D) GPY/F module multiple alignments between *Bel/Pao *and *Ty3/Gypsy *LTR retroelements. Click here for file

Additional file 3 **Network markers and graphs**. Excel file containing five sections; AF3A) guide file summarizing all markers; AF3B) bipartite multigraphs between distributions and PAM-like markers; AF3C) bipartite multigraphs between retroelement phylogeny and PAM-like markers; AF3D) summary of non-redundant MCs; AF3E) Phenotypic neighbors' network. Click here for file

Additional file 4 **Building multigraphs**. Zip-file containing all notebooks (Mathematica files) needed to visualize or reproduce graphs shown in this study. This is presented as a mini-web site containing three folders and two HTML files. Opening the HTML file called "Index.html" and following the steps summarized therein users can reproduce the analyses using Mathematica 7.0 or simply visualize them using the freely available Mathematica Player. Click here for file
