# Supplementary figures and images for: Network dynamics of eukaryotic LTR retroelements beyond phylogenetic trees
Source: Biol Direct. 2009 Nov 2;4:41. doi: 10.1186/1745-6150-4-41 (PMC2774666; doi:10.1186/1745-6150-4-41)

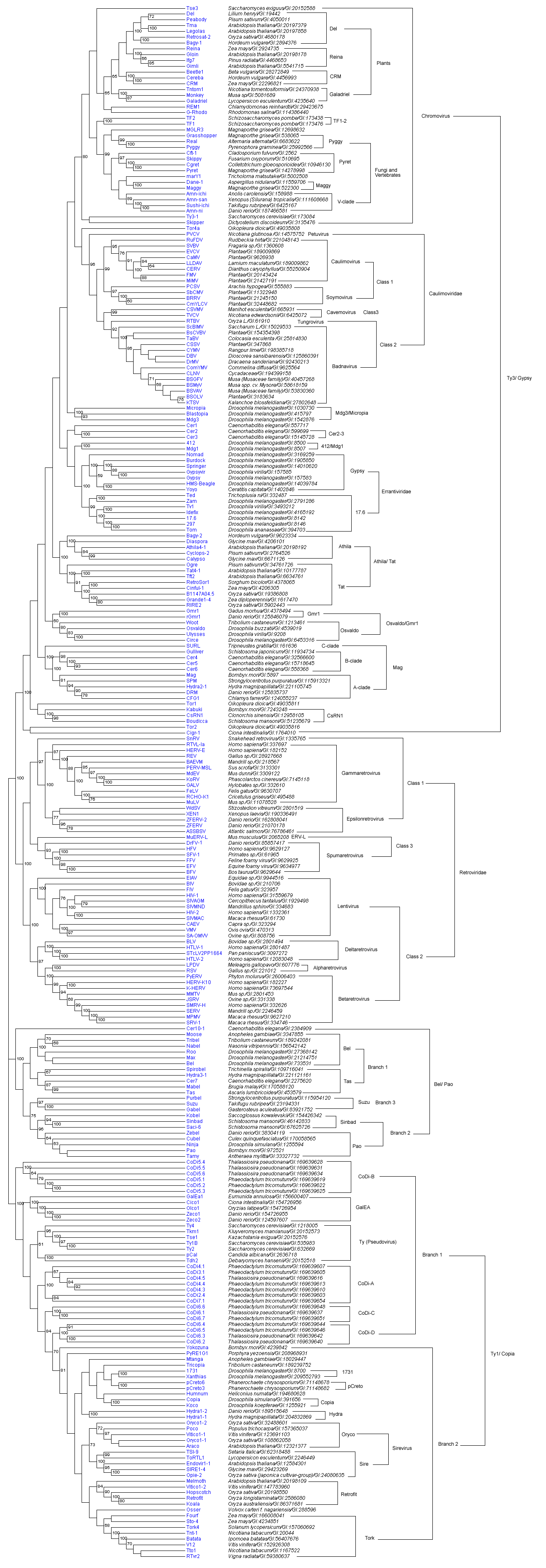

Supplement: Additional file 1 — LTR retroelement phylogeny. Inferred based on pol using the 268 LTR retroelements used in this study. This tree includes information about names, Genbank accessions and hosts of all LTR retroelement taxa used. By clicking on each OTU in this tree, the user can download a GyDB file or Genbank accession of the requested element from GyDB or NCBI, respectively. [file 1745-6150-4-41-S1.zip › Additional_file_1/AF1.png]

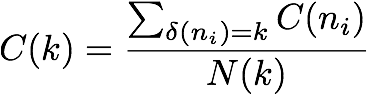

Supplement: Additional file 4 — Building multigraphs. Zip-file containing all notebooks (Mathematica files) needed to visualize or reproduce graphs shown in this study. This is presented as a mini-web site containing three folders and two HTML files. Opening the HTML file called "Index.html" and following the steps summarized therein users can reproduce the analyses using Mathematica 7.0 or simply visualize them using the freely available Mathematica Player. [file 1745-6150-4-41-S4.zip › Additional_file_4/images/formulae/C(k).png]

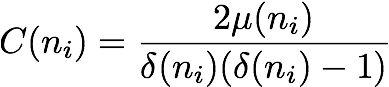

Supplement: Additional file 4 — Building multigraphs. Zip-file containing all notebooks (Mathematica files) needed to visualize or reproduce graphs shown in this study. This is presented as a mini-web site containing three folders and two HTML files. Opening the HTML file called "Index.html" and following the steps summarized therein users can reproduce the analyses using Mathematica 7.0 or simply visualize them using the freely available Mathematica Player. [file 1745-6150-4-41-S4.zip › Additional_file_4/images/formulae/C(n).png]

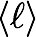

Supplement: Additional file 4 — Building multigraphs. Zip-file containing all notebooks (Mathematica files) needed to visualize or reproduce graphs shown in this study. This is presented as a mini-web site containing three folders and two HTML files. Opening the HTML file called "Index.html" and following the steps summarized therein users can reproduce the analyses using Mathematica 7.0 or simply visualize them using the freely available Mathematica Player. [file 1745-6150-4-41-S4.zip › Additional_file_4/images/formulae/mean(l).png]

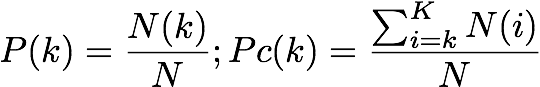

Supplement: Additional file 4 — Building multigraphs. Zip-file containing all notebooks (Mathematica files) needed to visualize or reproduce graphs shown in this study. This is presented as a mini-web site containing three folders and two HTML files. Opening the HTML file called "Index.html" and following the steps summarized therein users can reproduce the analyses using Mathematica 7.0 or simply visualize them using the freely available Mathematica Player. [file 1745-6150-4-41-S4.zip › Additional_file_4/images/formulae/P(k) - Pc(k).png]

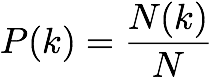

Supplement: Additional file 4 — Building multigraphs. Zip-file containing all notebooks (Mathematica files) needed to visualize or reproduce graphs shown in this study. This is presented as a mini-web site containing three folders and two HTML files. Opening the HTML file called "Index.html" and following the steps summarized therein users can reproduce the analyses using Mathematica 7.0 or simply visualize them using the freely available Mathematica Player. [file 1745-6150-4-41-S4.zip › Additional_file_4/images/formulae/P(k).png]

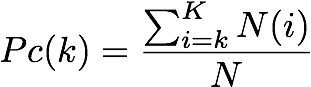

Supplement: Additional file 4 — Building multigraphs. Zip-file containing all notebooks (Mathematica files) needed to visualize or reproduce graphs shown in this study. This is presented as a mini-web site containing three folders and two HTML files. Opening the HTML file called "Index.html" and following the steps summarized therein users can reproduce the analyses using Mathematica 7.0 or simply visualize them using the freely available Mathematica Player. [file 1745-6150-4-41-S4.zip › Additional_file_4/images/formulae/Pc(k).png]
