# Supplementary material for: Network dynamics of eukaryotic LTR retroelements beyond phylogenetic trees
Source: Biol Direct. 2009 Nov 2;4:41. doi: 10.1186/1745-6150-4-41 (PMC2774666; doi:10.1186/1745-6150-4-41)
Supplement: Additional file 4 — Building multigraphs. Zip-file containing all notebooks (Mathematica files) needed to visualize or reproduce graphs shown in this study. This is presented as a mini-web site containing three folders and two HTML files. Opening the HTML file called "Index.html" and following the steps summarized therein users can reproduce the analyses using Mathematica 7.0 or simply visualize them using the freely available Mathematica Player. [file 1745-6150-4-41-S4.zip › Additional_file_4/index.html]

Additional file


# Additional file 4. Network construction and analysis with *Mathematica*

What follows is a step-by-step outline of the network construction and analysis methods performed in
*Mathematica*. Each section corresponds to each of the logical tasks carried out to construct and analyze the different network models. Sections are structured in three parts:

- Link to a Mathematica notebook.
- Short description of the notebook's contents.
- "Further details" link, which presents a detailed table with an ordered list of the operations and
  algorithms, as well as input and output data formats.

All notebooks can be viewed with the free *Mathematica Player*. Alternatively *Mathematica Player Pro* or
*Mathematica*

may also be used.

### 1. Excel workbook import

**01 - Excel data import.nb** (Download *Mathematica Player* to open notebooks)

This notebook imports the data provided in Additional File 3 (sheets AF3A and AF3D) into
*Mathematica*.

Further details

### 2. Hosts distribution-Markers bimultigraphs shown in Additional file 3 AF3B

**02 -
Distributions-Markers bimultigraphs.nb**  
(*Mathematica Player* or above required)

Builds and displays bimultigraphs which relate the different states of three polymorphic amino acid motifs (PAMs) present in retroelements with their host distributions.

Further details

### 3. Retroelement phylogeny-Markers bimultigraphs shown in Additional file 3 AF3C

**03 -
Branches-Markers bimultigraphs.nb**  
(*Mathematica Player* or above required)

Builds and displays bimultigraphs which relate the different states of three polymorphic amino acid motifs (PAMs) present in retroelement lineages with their branch groups.

Further details

### 4. Constructing phenotypic neighbors networks

**04a - MCs
feature vectors builder.nb**   
**04b - Phenotypic neighbors network.nb**  
(*Mathematica Player* or above required)

Creates a list of non-redundant feature vectors from 8 PAMs and constructs a network on the basis of
vector distances considering trait changes as edges (linking phenotypic neighbors). This network is
presented as a multigraph taking all lineage changes between two MCs as multiple edges.

Further details

### 5. Phenotypic neighbors network analyses (degree distribution, average clustering coefficient and mean shortest path length)

**05a - Phenotypic neighbors network analysis.nb**  

**05b - Phenotypic neighbors network analysis (lineage multiedges).nb**  
(*Mathematica Player* or above required)

The networks in the previous section are transformed into adjacency matrices and their degree distribution,
average clustering coefficient and mean shortest path length are calculated.

Further details
