# Supplementary material for: Network dynamics of eukaryotic LTR retroelements beyond phylogenetic trees
Source: Biol Direct. 2009 Nov 2;4:41. doi: 10.1186/1745-6150-4-41 (PMC2774666; doi:10.1186/1745-6150-4-41)
Supplement: Additional file 4 — Building multigraphs. Zip-file containing all notebooks (Mathematica files) needed to visualize or reproduce graphs shown in this study. This is presented as a mini-web site containing three folders and two HTML files. Opening the HTML file called "Index.html" and following the steps summarized therein users can reproduce the analyses using Mathematica 7.0 or simply visualize them using the freely available Mathematica Player. [file 1745-6150-4-41-S4.zip › Additional_file_4/table.html]

Additional file


Table 1. Excel workbook import

| Import Excel workbook in Additional File 3 into Mathematica | |
| 1.1 | Import sheet 1, AF3A, (rows 1-268) and sheet 4, AF3D, (rows 1-77). |
| 1.2 | Remove blank text cells from AF3D. |

Back to index

Table 2. Host distribution-Markers bimultigraphs (Additional file 3, AF3B)

| Build and display Host distributions-PAMs bimultigraphs | |
| 2.1 | Load module **buildBimultigraphNetwork** Arguments:  Tabular input data and three headers in the data which indicate, respectively, two bipartite sets of nodes and a set of edge labels.   Algorithm outline:  Obtain the column numbers for the second, third and fourth arguments. Traverse the input data, row by row, adding an edge for each row which joins the elements at the columns specified in the second and third arguments. Label the new edge with the element appearing at the fourth argument column of that row.   Returns:  List of edges. |
| 2.2 | For each marker "CCHC array", "P3 consensus", "GPY/F":  - Run buildBimultigraphNetwork(“excelAF3A”, “Distribution”, marker,   “Species”) - Plot graph returned by **buildBimultigraphNetwork** module |

Back to index

Table 3. Retroelement phylogeny-Markers bimultigraphs (Additional file 3, AF3C)

| Build and display retroelement phylogeny branches-PAMs bimultigraphs | |
| 3.1 | Load module **buildBimultigraphNetwork**. Arguments:  Tabular input data and three headers in the data which indicate, respectively, two bipartite sets of nodes and a set of edge labels.   Algorithm outline:  Obtain the column numbers for the second, third and fourth arguments. Traverse the input data, row by row, adding an edge for each row which joins the elements at the columns specified in the second and third arguments. Label the new edge with the element appearing at the fourth argument column of that row.   Returns:  List of edges. |
| 3.2 | For each marker "CCHC array", "P3 consensus", "GPY/F":  - Run buildBimultigraphNetwork (“excelAF3A”, “Branch”, marker,   “Lineage”) - Plot graph returned by **buildBimultigraphNetwork** module |

Back to index

Table 4. Constructing networks of phenotypic neighbors

| Construct MCs as feature vectors: MC → 8-dimensional integer vector | |
| 4.1 | Load auxiliary module **featureMap**. |
| 4.2 | Load main module **featureVectors**. Arguments:  Tabular data, column to use as key for each vector and names of features.   Algorithm outline:  Call **featureMap** to get a mapping which assigns each PAM state found to an integer value. For each element in the key column the set of features (third argument) is substituted according to the map previously obtained.   Returns:  Feature vectors and mapping used. |
| 4.3 | Assign to featureList a list of PAMs to use as they appear in the header of AF3A and run {fvs, fm} = featureVectors[excelAF3A, "Species", featureList]. |
| 4.4 | Remove duplicate MCs from fvs to get a unique list of 76 feature vectors. |
| Join MCs in a network according to PAM state changes | |
| 4.5 | Load auxiliary module **similarityMatrix**. |
| 4.6 | Load main module **buildDistanceGraph**. Arguments:  List of unique feature vectors of the same dimension. Boolean value to specify whether to stop after a first pass or continue until joining all vertices   Algorithm outline:  Use **similarityMatrix** in order to get a square matrix with the number of differences of all pairwise comparisons of the vectors. Starting with each vector as a one-vertex connected component, compare it with the vertices of the other components, joining two nodes if the number of differences is 1. Proceed equally with the rest of the connected components. Increase the number of differences until all nodes are in one component.   Returns:  List of edges. |
| 4.7 | Load auxiliary module **addVirtualNodes**. Algorithm outline:  For each edge in the feature vector distance graph, insert nodes proportionally to the number of differences between all nodes that differ in more than one change. |
| 4.8 | Plot graph returned by addVirtualNodes[buildDistanceGraph[Sort[Union[fvs]],True]] with visualization modules **setEdgeColor** and **setVertex**. |
| 4.9 | Plot graph returned by addVirtualNodes[buildDistanceGraph[Sort[Union[fvs]],False]] with visualization modules **setEdgeColor** and **setVertex**. |
| 4.10 | Load auxiliary module **addMultiLabeledEdges**. Algorithm outline:  For each edge joining two feature vectors (2 or 3 in the case of more than one change between them) add as many edges as different lineage changes share the MC. Label the edges accordingly. |
| 4.11 | Plot graph returned by  addMultiLabeldEdges[addVirtualNodes[buildDistanceGraph [Sort[Union[fvs]],False]]] with visualization modules **setEdgeColor2** and **setVertex**. |

Back to index

Table 5. Phenotypic neighbors network analyses

| Computation of degree distribution and average clustering coefficient of phenotypic neighbors networks | |
| 5.1 | Obtain adjacency matrix |
| 5.2 | Transform graph into *Combinatorica* format from adjacency matrix |
| 5.3 | Load module **degreeDistribution**. Arguments:  Graph (in *Combinatorica* format)   Returns:  Probability degree distribution,  *P*(*k*) , and cumulative probability degree distribution,  *Pc*(*k*)  for all degrees  *k*  in input graph.   Where *N*(*i*) → Nodes of degree *i* ; *N* → Number of nodes ; *K* → Maximum degree |
| 5.4 | Run degreeDistribution(graph). |
| 5.5 | Load auxiliary module **clusteringCoefficient**. Arguments:  Graph (in *Combinatorica* format) and node number   Returns:  Clustering coefficient of specified node,  *C*(*n*) .    Where *δ*(*ni*) →  Degree of node *ni* ; *μ*(*ni*) →  Number of edges between distance 1 neighbors of *ni* |
| 5.6 | Load main module **averageClusteringCoefficients**. Arguments:  Graph (in *Combinatorica* format).   Returns:  Clustering coefficient  *C*(*k*)  for all degrees  *k*  in input graph. |
| 5.7 | Run averageClusteringCoefficients(graph). |
| 5.8 | Calculate the mean shortest path length :  Run Mean[Mean[AllPairsShortestPath[graph]]] |

Back to index
